# Complementary intra- and extracellular AGR2 activities support epithelial ovarian cancer cell aggressiveness

**DOI:** 10.1038/s41388-026-03938-y

**Published:** 2026-08-10

**Authors:** Marianne Guilbard, Saïd Taouji, Marc Aubry, Jacques Robert, Raphael Pineau, Valérie Velasco, Romane Florent, Louis-Bastien Weiswald, Cecile Hartog, Coriolan Lebreton, Alix Coulon, Sabrina Croce, Guillaume Babin, Cédric Chaveroux, Stéphanie Beq, Eric Chevet, Delphine Fessart, Frédéric Delom

**Affiliations:** 1 OncoGyne Lab, Université de Bordeaux, Institut Bergonié, Inserm, Bordeaux, France; 2 Thabor Therapeutics, Paris, France; 3 INSERM U1242, University of Rennes, Rennes, France; 4 Centre de Lutte contre le cancer Eugène Marquis, Rennes, France; 5 Department of Biopathology, Institute Bergonié, Comprehensive Cancer Centre, Bordeaux, France; 6 Université de Caen Normandie, PLATON Services Unit, ORGAPRED core facility, Caen, France; 7 Université de Caen Normandie, INSERM U1086 ANTICIPE (Interdisciplinary Research Unit for Cancers Prevention and Treatment), Caen, France; 8 CNRS - LBTI, UMR 5305 - Université Claude Bernard Lyon 1, Institut de Biologie et Chimie des Protéines, Lyon, France

**Keywords:** Endoplasmic reticulum, Ovarian cancer

## Abstract

Anterior Gradient 2 (AGR2) is an endoplasmic reticulum (ER)-resident protein that belongs to the protein disulphide isomerase (PDI) family, and whose expression and secretion are induced by stress. Extracellular (secreted) AGR2 has been proposed as a marker of ER stress–related proteostasis alterations. Cancer cells frequently overexpress intracellular AGR2 (iAGR2) and secrete extracellular AGR2 (eAGR2). These features are associated with tumour progression and may serve as potential biomarkers in epithelial ovarian cancer (EOC). To investigate the roles of both iAGR2 and eAGR2 in EOC, we first generated EOC cells overexpressing iAGR2 and secreting eAGR2. Antibodies blocking eAGR2 reduced the proliferation and migration of these overexpressing cells. Concurrently, supplementation of parental cells with recombinant eAGR2 partially rescued these properties, further supporting a functional extracellular role for AGR2 in EOC. Quantitative proteomics, complemented by analysis of the TCGA database, revealed that eAGR2 modulated the expression of proteins involved in autophagy. This suggests that eAGR2-induced signalling may enhance catabolic activity under stress conditions, thereby increasing nutrient availability and, in turn, facilitating protein synthesis. This was reflected in the increased translational activity observed in AGR2-overexpressing and eAGR2-stimulated cells. Our results highlight two distinct, compartmentalised roles for AGR2. Specifically, iAGR2 acts as an ER-resident PDI, enhancing protein folding and ER quality control. In a complementary manner, eAGR2 functions as a metabolic regulator that may relieve constraints on tumour cell aggressiveness by maintaining autophagic flux and promoting protein synthesis. Overall, these findings support a dual-compartment model in which iAGR2 couples ER proteostasis with the metabolic and translational stimulation mediated by eAGR2.

## Introduction

Ovarian cancer is the fourth leading cause of cancer-related mortality in women worldwide and remains the most lethal gynaecological malignancy (GLOBOCAN 2022, IARC). Its poor prognosis is largely due to the fact that most cases are diagnosed at advanced stages. Although initial responses to cytoreductive surgery combined with platinum–taxane chemotherapy are high, most patients experience relapse. This relapse is frequently associated with chemoresistance [1, 2]. The vast majority of epithelial ovarian tumours (EOC) exhibit genomic instability, extensive chromosomal aberrations, and frequent defects in homologous recombination repair [3].

Genomic instability is a well-established hallmark of cancer cells [4]. Intrinsic genomic instability can increase the load of misfolded proteins in the Endoplasmic Reticulum (ER), leading to elevated ER stress [5]. Cancer cells, owing to their high mutational burden, depend heavily on the UPR for survival. Samanta et al. [6] suggested that the increased expression of ER stress-associated proteins in EOC indicates a role for ER stress and the UPR in this cancer type (EOC). A critical component of this adaptive response is the upregulation of protein disulphide isomerases (PDIs), a family of ER-resident proteins that assist protein folding by catalysing disulphide bond formation and rearrangement [7, 8]. By alleviating the burden of misfolded proteins, PDIs mitigate ER stress and allow cancer cells to evade cell death and survive [9]. The upregulation of PDI expression is a common feature of many cancers [10], reflecting their essential role in accommodating the heightened demand for protein folding in rapidly dividing tumour cells. Moreover, the expression of PDI family members has been shown to correlate positively with tumour progression and negatively with patient survival in EOC [6, 11].

Among the various protein disulphide isomerases (PDIs) involved in cancer, Anterior Gradient 2 (AGR2), also known as PDIA17, is frequently overexpressed in EOC [11]. AGR2 primarily functions in the ER by catalysing disulphide bond formation, ensuring proper folding of nascent proteins, and maintaining protein quality control and intracellular homeostasis in cancer cells [8]. We and others have linked AGR2 to ER stress and activation of the unfolded protein response (UPR) [10, 12, 13]. The UPR mitigates stress by reducing the protein load, enhancing ER folding capacity, or removing misfolded proteins [14]. Persistent accumulation of improperly folded proteins causes proteotoxic stress [15], dysregulating survival and death pathways [16]. We demonstrated that two key UPR sensors, inositol-requiring enzyme 1 (IRE1) and activating transcription factor 6 (ATF6), modulate AGR2 expression [12]. AGR2 localisation in the ER is regulated by dimerization, which is disrupted under ER stress, leading to AGR2 secretion [17]. Extracellular AGR2 can induce epithelial-to-mesenchymal transition (EMT) in an autocrine manner or attract monocytes [17, 18]. Thus, AGR2 exists both intracellularly (iAGR2) and extracellularly (eAGR2), coordinating protein folding inside the cell and signalling or modulatory functions outside. In addition, AGR2 has been consistently and through different mechanisms associated with several features of cancer [19, 20].

Extracellular AGR2 (eAGR2) is detectable in the serum of EOC patients [21, 22], with levels significantly higher than in healthy people [23]. Integrated into multi-marker panels including CA125, HE4, MDK, and AGR2, eAGR2 improves longitudinal detection models, as shown in the UKCTOCS trial [24]. In mice bearing ovarian cancer cells xenografts, a humanised antibody targeting eAGR2 reduces tumour growth [25], and hypomethylation of the AGR2 promoter correlates with invasive and metastatic EOC phenotypes [26]. Functionally, AGR2 overexpression in EOC was shown to enhance proliferation and migration and up-regulates genes involved in cell growth, invasion, and angiogenesis, thereby promoting tumour progression and metastatic potential [27]. However, it remains unclear whether these effects originate from intracellular AGR2 or extracellular AGR2, leaving the specific contribution of eAGR2 to EOC progression to be determined. While evidence in EOC remains limited, experimental studies in other epithelial tumour models have shown that eAGR2 can promote tumour cell aggressiveness (proliferation, migration and invasion) [18, 26, 28–31].

Herein we hypothesised that eAGR2 could contribute to tumour cell aggressiveness. To test this hypothesis, we investigated the role of eAGR2 by altering its extracellular availability with anti-AGR2 neutralizing antibodies or human AGR2 recombinant protein. Given the high histological and clinical heterogeneity of EOC, we used an approach combining controlled ‘gain-of-function’ cellular models, patient-derived tumour organoids (PDTOs), and large clinical cohorts to investigate the role of AGR2. Importantly, our results demonstrate that eAGR2 functions not only as a driver of tumour cell aggressiveness, promoting proliferation and migration, but also as a regulator of trafficking, autophagy, and protein synthesis. These observations support a bifunctional model of AGR2 in tumour cells. The intracellular fraction of AGR2 (iAGR2) contributes to the maintenance and enhancement of ER proteostasis. Meanwhile, the extracellular fraction of AGR2 (eAGR2) promotes metabolic activity and translation, thereby supporting the increased biosynthetic demands of tumour cells. Together, i- and eAGR2 act in a complementary manner, enabling tumour cells to manage proteotoxic stress while sustaining high biomolecular production requirements.

## Material and method

### Cell lines and culture

Human EOC cell lines, SKOV3 and OVCAR3, were cultured in RPMI 1640 and RPMI 1640 high-glucose medium, respectively, supplemented with 10% foetal bovine serum (FBS) and 1% penicillin-streptomycin. The SKOV3 medium was further supplemented with 1% sodium bicarbonate, while the OVCAR3 medium was supplemented with insulin (10 µg/mL). Human colorectal cancer cell line HCT116 was cultured in McCoy’s 5 A medium, supplemented with 10% FBS and 1% penicillin-streptomycin. Human lung cancer cell line A549 was cultured in DMEM supplemented with 10% FBS and 1% penicillin-streptomycin. All cell lines used for this study were mycoplasma-free. To overexpress AGR2, SKOV3 and OVCAR3 cells were infected with an empty pLenti6.3/V5 vector or a pLenti6.3/V5 vector bearing the coding sequence of human AGR2 [18] and selected with 5 µg/mL Blasticidine.

### Patient-Derived Tumour Organoid (PDTO) culture

Ovarian cancer patient-derived tumour organoids (PDTOs) were established and cryopreserved as previously described [32, 33]. Cryopreserved dissociated cells from seven PDTO models, stored in Recovery™ Cell Culture Freezing Medium (Gibco) at −150°C, were rapidly thawed in a 37 °C water bath. Subsequently, thawed cells were immediately transferred to pre-warmed organoid basal medium (OBM; Advanced DMEM supplemented with 10 U/mL penicillin, 10 µg/mL streptomycin, and 1% GlutaMAX-1; Fisher Scientific). Cells were pelleted by centrifugation at 430 g for 5 min at 4°C, resuspended in organoid culture medium, and embedded in 70% Cultrex Reduced Growth Factor Basement Membrane Extract, Type 2 (BME2; Trevigen). The organoid culture medium included OBM supplemented with B27 (200 µL/mL; Fisher Scientific), N-Acetyl-L-cysteine (1.25 mM; Sigma), EGF (50 ng/mL; Miltenyi), FGF10 (20 ng/mL; Peprotech), FGF-basic (1 ng/mL; Miltenyi), A-83-01 (500 nM; Peprotech), Y-27632 (10 µM; Selleckchem), SB202190 (1 µM; Peprotech), nicotinamide (10 mM; Sigma), PGE2 (1 µM; Sigma), Primocin (100 µg/mL; InvivoGen), R-spondin-1-Fc 293 T conditioned medium (50% v/v; AMS Bio), and L-WRN conditioned medium (10% v/v; AMS Bio). PDTO–BME2 suspensions (50 µL drops) were seeded in pre-warmed 24-well plates (Corning) or 6-well plates (Nunc). After polymerisation at 37 °C with 5% CO₂ for 15 min, each dome was overlaid with organoid culture medium and incubated in a humidified incubator (37 °C, 5% CO₂). Culture medium was replaced twice per week. Importantly, all PDTO lines were confirmed to be mycoplasma-free and authenticated by STR profiling (Microsynth) against their corresponding primary tumour samples. PDTOs were further characterised as previously described [32], and histological subtypes (high-grade serous carcinoma [HGSC] or clear cell carcinoma [CCC]) were validated by immunohistochemistry.

### Preparation of supernatant from PDTO cultures

When organoids reached approximately 150 µm in diameter, they were harvested using cold OBM supplemented with 1% bovine serum albumin (BSA). The organoids were centrifuged at 200 g for 2 min and incubated with TrypLE™ Express (Gibco) for up to 15 min at 37 °C. Following enzymatic dissociation, cells were centrifuged at 430 g for 5 min, resuspended in organoid culture medium, counted, and seeded at 10,000 cells per 50 µL drop of 70% BME2 in pre-warmed 24-well plates. After polymerisation (37 °C, 5% CO₂, 15 min), each dome was covered with organoid culture medium and maintained under standard culture conditions, with medium changes twice weekly. Once organoids reached 150–250 µm in diameter, fresh medium was added and cultures were incubated for 72 h. Finally, supernatants were collected, centrifuged at 430 g for 5 min to remove debris, and stored at −80°C until further analysis.

### Immunofluorescence microscopy

Cells were seeded on coverslips in 12wp at a density of 31,000 cells/cm^2^ and cultured for 48 h. Following incubation, cells were fixed with 4% paraformaldehyde (PFA) for 12 min at room temperature (RT), then permeabilized with 0.1% NP-40 in PBS 1X for 15 min. Subsequently, cells were incubated with primary antibodies diluted in blocking solution (0.5% Tween 20, 1% BSA, PBS 1X) for 1 h at RT. The following primary antibody was used: mouse monoclonal anti-AGR2 (clone 1C3, cat# H00010551-M03, Abnova). After washing with PBS 1X, cells were incubated with Alexa Fluor-conjugated secondary antibodies and DAPI (4′,6-diamidino-2-phenylindole) for nuclear staining for 1 h at RT in the dark. Coverslips were washed, mounted using antifade mounting medium, and analysed using a Leica inverted fluorescence microscope (DMi8 S Platform, Leica Microsystems).

### Western blot

Cells were lysed and then processed for immunoblot analysis as previously described [34]. Quantification was performed using ImageJ software. Results were normalized to the anti-Calnexin protein and are presented as fold change compared with the control, which was set to one. The signals were detected using an enhanced chemiluminescence detection kit (Millipore, #P90720). The sources of the primary antibodies used on this study are as follow: anti-AGR2 (17 kDa) (Abnova, #H00010551-M03), anti-GAPDH (36 kDa) (Merk, #MAB-374), anti-Calnexin (90 kDa) (Cell signaling, #2679), anti-SLCA711 (Cell Signalling, #12691), anti-VPS18 (Proteintech, #10901-1-AP), anti-PIK3C3 (Cell signalling, #4263). The secondary antibodies used were as follows: anti-Mouse (Cytiva, #NA031), anti-Rabbit (Cytiva, #NA9340-1ML).

### EMT status classification using CCLE transcriptomic data

Gene expression data from the Cancer Cell Line Encyclopaedia (CCLE) were used to assess epithelial–mesenchymal transition (EMT) status across cancer cell lines. EMT classification was based on a curated panel of 44 epithelial-associated genes and 33 mesenchymal-associated genes, as defined by Kohn et al. [35], representing key markers of epithelial and mesenchymal phenotypes. Raw read counts were log2-transformed and subsequently z-score normalized across samples to facilitate comparative analysis and reduce variability due to gene-specific expression ranges. All data processing and normalization were performed using RStudio.

### Preparation of cell pellets for histological analyses

Cells were harvested at 80% confluence in the presence or absence of recombinant AGR2 (eAGR2), washed twice with cold PBS 1X, and centrifuged at 300 g for 5 min to obtain a compact pellet. After fixation in 4% paraformaldehyde for 24 h at 4 °C, pellets were centrifuged at 1200 rpm for 5 min and embedded in 3% low-melting agarose. Agarose plugs were transferred into embedding cassettes, processed through routine dehydration, and paraffin-embedded. Paraffin blocks were sectioned into 4 µm slices using a microtome, mounted on glass slides, and subjected to immunohistochemistry (IHC).

### Immunohistochemistry and histopathological analyses

IHC staining was performed on paraffin sections using the Benchmark® ULTRA automated platform (Ventana, Roche). Detection was carried out with either the UltraView Universal DAB (760-500) or the OptiView DAB IHC (760-700) kits, depending on the antibody used. Optimization of each primary antibody included positive and negative controls to define dilution, antigen retrieval, and incubation conditions. Sections were visualized with a NikonEclipse501 microscope, and images were acquired using NIS-Elements F 3.00 System (Nikon Digital Sight). Antibody list: p53 (clone DO7, Roche Diagnostics, 760-4242), vimentin (clone V9, Roche Diagnostics GmbH, 790-2917), E-cadherin (clone 36, Roche Diagnostics GmbH, 790-4497), p16 (clone INK4a, Roche Diagnostics, 805-4713), and SOX2 (clone SP76, Roche Diagnostics, 760-4621).

### Quantification of immunohistochemical staining by digital image analyses

Cell pellets were stained with the antibody of interest, revealed using DAB chromogen (brown), and counterstained with haematoxylin (blue nuclei). Whole-slide images were acquired by brightfield scanning microscopy (Hamamatsu NDPI format) and analysed using QuPath (v0.7.0). For each condition, a region of interest (ROI) was manually defined on the corresponding cell pellet. Staining quantification was performed using the Positive Cell Detection module: individual nuclei were detected on the Haematoxylin OD channel, and DAB positivity was assessed using the Cell DAB OD mean for cytoplasmic and membranous markers (E-cadherin and vimentin) or the Nucleus DAB OD mean for strictly nuclear markers (SOX2). Cells were classified as DAB-positive using a single threshold of 0.2 OD units. This threshold (typically 0.2 OD units) was determined for each marker based on negative control slides to ensure optimal sensitivity and specificity. Furthermore, automated detections were visually validated by two experienced pathologists (AC and SC) to ensure accuracy. Results are expressed as the percentage of DAB-positive cells relative to the total number of detected cells within each ROI, providing a normalised measure of protein expression independent of ROI size and local cell density.

### Reverse transcription (RT)-PCR and real-time quantitative PCR

Cells were seeded in six-well plates at a density of 16,000 cells/cm², and total RNA was extracted using the RNeasy Mini Kit (Qiagen). One microgram of total RNA was reverse-transcribed to cDNA using the SuperScript Vilo master mix (Thermofisher). Real-time PCR was performed using the Luna universal master Mix (New England BioLab) on a One-Step Plus Real-time PCR system (Applied Biosystems). Real-time RT-PCR profile consists of 10 min of initial activation at 95 °C followed by 40 cycles of 15 s denaturation at 95 °C, and 1 min annealing and extension at 60 °C and an association stage including 15 s at 95 °C, 1 min at 60 °C and 15 s at 95 °C. The following gene-specific primers were used: *AGR2* forward (5’-ATGAGTGCCCACACAGTCAA-3’), *AGR2* reverse (5’-GGACATACTGGCCATCAGGA-3’); *GAPDH* forward (5’-CCTGGTATGACAACGAATTT-3’), and *GAPDH* reverse (5’-GTGAGGGTCTCTCTCTTCCT-3’), as previously described [26]. Relative gene expression levels were calculated using the ΔΔCt method, with *GAPDH* serving as the reference gene for normalization.

### Quantification of AGR2 in the extracellular medium using Gyrolab® automated immunoassays

Cells were seeded in 6-well plates at a density of 16,000 cells/cm². After 72 h of incubation, the conditioned medium was collected, centrifuged at 1200 rpm for 5 min to remove cellular debris, and passed through a 0.22 µm filter. Extracellular AGR2 levels were quantified using the Gyrolab® automated immunoassay platform (Gyros Protein Technologies). Fluorescence signals, proportional to target protein concentration, were acquired and analysed using Gyrolab® software. Protein concentrations in the samples were determined by interpolation from a standard curve generated with known AGR2 concentrations.

### Proliferation assays

Proliferation assays were performed on the POETIC platform. Cells were seeded into 96-well plates at a density of 7000 cells/cm^2^, and proliferation was monitored over 120 h by quantifying DNA content with propidium iodide (PI). At the indicated time points, cells were fixed with 4% paraformaldehyde, treated with RNase A (10 µg/mL, 2 h, 37 °C), and stained with PI (12.5 µg/mL, 10 min, RT). Fluorescence was measured on an EnVision plate reader (Ex 544 nm/Em 615 nm). The PI signal was used as a proxy for cell number, and proliferation was expressed as fold change relative to the 24 h control condition.

### Cellular migration by scratch assays

Cells were seeded in 24-well plates at a density of 84,000 cell/cm^2^ and cultured until reaching ~80–90% confluence. Using a sterile pipette tip, a straight scratch was made across the cell monolayer in each well. Detached cells were removed by gently washing the wells twice with PBS 1X. Following the scratch (0 h), AGR2 blocking antibody or an IgG non-relevant isotype antibody (both at 40 µg/mL) were added to the wells, and cells were incubated in serum-free medium. Wound closure was monitored over a 48-hour period. Images of the scratch area were acquired at 0 h and at regular intervals (24, 48 h) using the DMi8 S Platform microscope (Leica) equipped with an HC PL APO 10x/0.40 DRY UV objective and a Hamamatsu C11400-22CU camera. The images were acquired using the LasX software (Leica Microsystems). The width of the scratch was measured using ImageJ software with the Wound Healing Tool macro (RRID:SCR_025260).

### Antibody-mediated eAGR2 neutralization

Proliferation Assay: Cells were seeded in 96-well plates at a density of 7000 cells/cm² and allowed to adhere for 24 h. The medium was then removed, cells were washed once with PBS 1X, and cultured in low-serum medium (5% FBS). Cells were treated with either 40 µg/mL of an AGR2-blocking antibody (Ab-AGR2; mouse monoclonal, clone 1C3, cat# H00010551-M03, Abnova) or a non-specific isotype control antibody (IgG control). After 72 h of treatment, proliferation was quantified as described above. Migration Assay: Cells were seeded in 24-well plates at a density of 84,000 cells/cm² and allowed to adhere for 24 h. The medium was then removed, cells were washed once with PBS 1X, and cultured in serum-free medium. Cells were treated with either 40 µg/mL of an AGR2-blocking antibody (eAb-AGR2) or a non-specific isotype control antibody (IgG control) for 72 h. After 48 h incubation, migration was assessed as described above.

### Production of recombinant human AGR2

Recombinant GST fusion AGR2 proteins were expressed in Escherichia coli (DH5α) and purified as previously described [36] with minor modifications. The recombinant AGR2-GST protein was purified using a GSTrap 4B affinity column (Cytiva, #29058804).

### Recombinant AGR2 treatments

#### Proliferation Assay

Cells were seeded in 96-well plates at a density of 7000 cells/cm² and allowed to adhere for 24 h. The medium was then removed, cells were washed with PBS 1X, and cultured in low-serum medium (5% FBS). Recombinant AGR2 was added at a final concentration of 200 ng/mL. Cells were re-challenged every 24 h, and a complete medium change was performed 48 h after the first stimulation. After 72 h of recombinant AGR2 exposure, proliferation was quantified as described above.

#### Migration Assay

Cells were seeded in 24-well plates at a density of 84,000 cells/cm² and allowed to adhere for 24 h. The medium was then removed, cells were washed with PBS 1X, and cultured in serum-free medium. Recombinant AGR2 was added at a final concentration of 200 ng/mL. Cells were re-challenged every 24 h with the same concentration. After 48 h of recombinant AGR2 exposure, migration was evaluated as described above.

### Sample preparation for MS analyses

SKOV3-AGR2 cells were seeded in 12wp at a density of 7800 cell/cm^2^. After 24 h cell seeding cells were treated with either AGR2-blocking antibody (eAb-AGR2) or an isotype control (IgG) for 72 hours. Following treatment, proliferation was assessed, and cells were lysed (20 mM Tris HCl, 1.5% CHAPS, 150 mM NaCl, QSP water, 1 mM Phosphatase inhibitors (NaF and Na_3_VO_4_), 10X Protease inhibitors). 50 or 60 µL of protein samples were mixed with lysis buffer provided in the easyPep mini-Kit (Thermo Scientific) to obtained a final volume of 100 µL. The samples were reduced and alkylated by incubation at 95°C for 10 min and continuously shaken at 1000 rpm. Then samples were digested with LysC/Trypsin mixture enzyme for 3 h at 37°C and processed following the manufacturer’s protocol. After a cleaning step, peptides were dried, suspended in 0.1% Formic Acids and dosed with the quantitative fluorometric peptide assay (ref. 23290, Thermo Scientific).

### NanoLC-MS/MS analyses

100 ng of each sample were analysed on the Exploris 480 mass spectrometer coupled with a Vanquish NEO nanoLC system (Thermo Scientific). Peptides samples were loaded on a C18 Acclaim PepMap100 trap-column 300 µm ID x 5 mm, 5 µm, 100 Å (ThermoFisher Scientific) and separated on a easyspray C18 Acclaim Pepmap100 nano-column, 50 cm×75 µm i.d, 2 µm, 100 Å (Thermo Scientific) with a 22.5 minutes linear gradient from 3% to 25% buffer B (A: 0.1% FA in H_2_O, B: 0.1% FA in ACN/H_2_O (80/20)) from 25% to 35% of B in 7.5 min and then from 35% to 100% of B in 0.1 min, hold for 12 min and followed by a washing and equilibration steps for a total duration of 50 min. The flow rate was 300 nL/min and the column temperature was kept constant at 45°C. Peptides were analysed with a DDA 1 s HCD method: MS data were acquired in a data dependent strategy (DDA) selecting the fragmentation events based on the most abundant precursor ions in a 1 s survey scan (350-1400 Th). Resolutions of the survey and MS/MS scans were respectively set at 120,000 and 15,000 at m/z 200 Th. The Ion Target Values for the survey and the MS/MS scans in the Orbitrap were set to 3E6 (300%) and 1E5 (100%) respectively and the maximum injection time was set to 50 ms for MS scan and 22 ms for MS/MS scan. Parameters for acquiring HCD MS/MS spectra were as follows: collision energy = 30 and isolation window = 4 m/z. The precursors with unknown charge state, charge state of 1 and 6 or greater than 6 were excluded. Peptides selected for MS/MS acquisition were then placed on an exclusion list for 40 s using the dynamic exclusion mode to limit duplicate spectra.

### Mass spectrometry data processing and quantification

Raw data were processed with Proteome Discoverer 3.1 (Thermo Scientific) through the Chimerys search engine against the SwissProt Homo sapiens database (Release march 2025, 20343 sequences). Precursor mass tolerance was set at 10 ppm and fragment mass tolerance was set at 0.02 Da, and up to 2 missed cleavages were allowed. Oxidation (M), phosphorylation (S, T, Y), acetylation (N-ter protein) was set as variable modification and Carbamidomethylation (C) as fixed modification. Validation of identified peptides and proteins was done using a target decoy approach with a false discovery rate positive (FDR < 1%) via Percolator. Protein quantitation was performed with precursor ions quantifier node in Proteome Discoverer 3.1 software. Proteins were normalized to the total peptide amount and ratios of quantitation were calculated in a pairwise way. Statistical validation was based on t-test.

### eAGR2 network construction

To investigate the molecular interactions of extracellular AGR2 (eAGR2), we constructed a protein–protein interaction network using Cytoscape (version 3.10.4, Cytoscape Consortium, USA). Proteomic eAGR2 modulators were retrieved from publicly available databases, including STRING and BioGRID. The dataset of eAGR2-associated proteins was imported into Cytoscape, and the network was visualised using the Prefuse Force Directed layout, which optimises node distribution for clarity. In this network, nodes represent proteins, while edges represent interactions. Key hub proteins were identified based on high degree and betweenness centrality values. Furthermore, functional enrichment analysis of network clusters was performed using the ClueGO plugin, linking proteins to Gene Ontology (GO) terms as well as biological processes and cellular components.

### TCGA-based transcriptomic and correlation analysis

RNA-seq data from The Cancer Genome Atlas (TCGA) were extracted for three tumour types: ovarian serous cystadenocarcinomas (OV), colon adenocarcinomas (COAD), lung adenocarcinomas (LUAD). Differential expression values were normalised using the Trimmed Mean of M-values (TMM) method [37]. Pearson correlation coefficients and associated p-values were computed between AGR2 and each gene of interest, independently within each cancer type. Analyses were performed in R (v4.5.1) using the cor.test function, applying Pearson’s method to assess linear relationships. The results were combined into a single dataset and annotated with correlation strength and statistical significance for downstream comparative analysis.

### SUrface SEnsing of Translation (SUnSET assay)

To assess global protein synthesis, the SUnSET assay was performed as previously described [38]. In brief, cells were seeded in 6-well plates at density of 16,000 cell/cm^2^ and treated with 5 µg/mL puromycin for 15 minutes at 37°C once they reached 75–80% confluence. Following a rinse with warm medium, cells were immediately lysed (20 mM Tris HCl, 1.5% CHAPS, 150 mM NaCl, QSP water, 1 mM Phosphatase inhibitors (NaF & Na_3_VO_4_), 10X Protease inhibitors). Immunoblot analysis was performed as previously described in [38]. Puromycylated peptides were detected using an anti-puromycin antibody (Merck, #MABE343).

### Autophagic flux inhibition assays

Cells were seeded and allowed to adhere for 24 h before treatment with 200 ng/mL recombinant AGR2. This treatment was replenished daily over a 72 h period. During the final 24 h, 150 nM Bafilomycin A1 (BafA1) was added to inhibit autophagic flux. Finally, cell proliferation, protein expression, and protein synthesis were assessed as described above.

### Publicly available datasets

Transcriptomic data were retrieved from publicly available datasets. TCGA RNA-seq data were obtained through the Genomic Data Commons (GDC) portal (https://portal.gdc.cancer.gov/) for the following cohorts: ovarian serous cystadenocarcinomas (OV), lung adenocarcinomas (LUAD), and colon adenocarcinomas (COAD). For comparison with normal ovarian tissue, RNA-seq data were retrieved from the Genotype-Tissue Expression (GTEx) project (https://gtexportal.org/). Proteomic data from TCGA OV were obtained and processed as previously described by Zhang et al. [39], providing a comprehensive resource for downstream analyses. All datasets used in this study are publicly available, and all cohort-level analyses were performed using RStudio.

## Results

### Histological and molecular characterisation of AGR2 expression in epithelial ovarian tumours

**Fig. 1:**
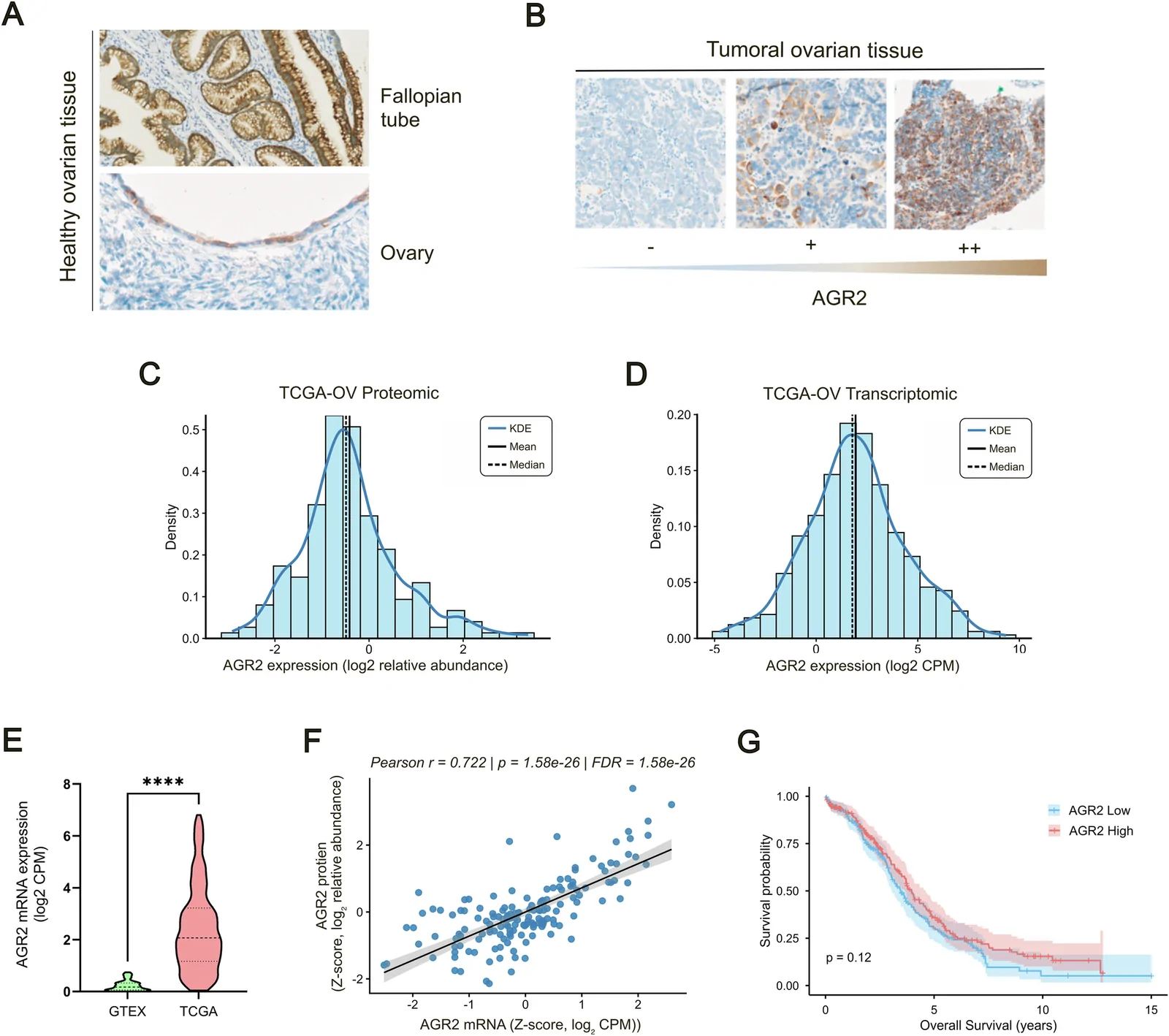
AGR2 and EOC. **A** AGR2 expression in healthy ovarian tissues was assessed by immunohistochemistry on formalin-fixed, paraffin-embedded sections of normal human ovary and fallopian tube (images acquired at 40× magnification). **B** A similar approach was performed on EOC tissues from our EOC cohort (n = 19). **C** Distribution of AGR2 expression TCGA-OV proteomic (n = 203; log₂ relative abundance normalized to a pooled reference) and **D** TCGA-OV transcriptomic (n = 417; log2 counts per million [CPM]) cohorts. Histograms display log₂-transformed expression values, with Kernel Density Estimation (KDE) curves applied to visualize probability density using a Gaussian kernel and default bandwidth. Mean (solid line) and median (dashed line) are indicated within each distribution. **E** Comparison of AGR2 mRNA expression levels between EOC (TCGA) and normal ovarian tissue (GTEX). Expression data were obtained from the TCGA-OV cohort (n = 417) and the GTEx cohort representing healthy ovarian tissue (n = 176). Values correspond to log₂ counts per million (log2 CPM) from normalized RNA-seq data. Statistical significance was determined using a two-tailed unpaired Welch’s t-test (**** *p* < 0.0001). **F** Pearson correlation between AGR2 mRNA and protein expression in the TCGA-OV cohort. Data were z-score normalized prior to analysis. Statistical significance was determined using Benjamini–Hochberg FDR-adjusted p-values (n = 157). **G** Kaplan–Meier analysis of overall survival in EOC patients from the TCGA-OV cohort (n = 415 patients). Patients were stratified into AGR2-high and AGR2-low groups according to the median AGR2 transcript level (median-based dichotomization, median= 1.78). Overall survival probabilities were estimated using the Kaplan–Meier method and compared using the log-rank test.

Immunohistochemical analysis of healthy ovarian tissue sections revealed AGR2 staining specifically restricted to the surface epithelium, with no detectable expression in the underlying medullary zone (Fig. 1A). Consistently, AGR2 was observed in the simple columnar epithelial layer of the Fallopian tube mucosa, whereas the underlying lamina propria remained negative (Fig. 1A). To evaluate AGR2 expression in ovarian tumours, we examined immunohistochemical staining of tumour sections from our local EOC cohort (n = 19, Table 1). This analysis revealed a heterogeneous staining pattern across tumour specimens, with AGR2 expression ranging from absent to strong, reflecting a continuous gradient (Fig. 1B, Table 1). This heterogeneity is further illustrated by representative images of AGR2-low and AGR2-high specimens from the Human Protein Atlas (Supplementary Fig. 1A). To complement these histological observations, AGR2 protein expression was analysed in the TCGA-OV proteomic cohort (*n* = 203). The histogram with density overlay (Fig. 1C) displayed a single, well-defined peak, consistent with a unimodal distribution of AGR2 protein levels. Statistical tests of multimodality, including Hartigan’s Dip (D = 0.017, *p* = 0.9747) and Silverman’s (*p* = 0.8643) tests, together with Gaussian mixture modelling (2 component), all supported the presence of a single continuous population (Table 2). A similar analysis of the TCGA-OV transcriptomic dataset (*n* = 417) revealed a comparable unimodal pattern of AGR2 protein abundance (Hartigan’s Dip D = 0.015, *p* = 0.8288; Silverman’s *p* = 0.9296; Gaussian mixture modelling 1 component) **(**Fig. 1D, Table 2). The unimodal yet broad distribution of AGR2 transcript and protein expression paralleled the inter-tumour variability observed by immunohistochemistry, indicating concordant heterogeneity at both molecular and tissue levels. Comparison between healthy ovarian tissues from the GTEx cohort (*n* = 176) and tumour samples from the TCGA-OV cohort (*n* = 417) showed significantly higher AGR2 mRNA levels in cancer tissues (Fig. 1E). Given the large sample sizes and the descriptive aim of cohort-level characterisation, TMM-normalised TCGA–GTEx comparisons offer an initial estimate of differential AGR2 expression. To further assess the relationship between mRNA and protein expression, paired transcriptomic and proteomic data from the TCGA-OV cohort (*n* = 157) were analysed (Fig. 1F). Kaplan–Meier survival analysis showed no significant difference in overall survival between patients categorised as AGR2-high versus AGR2-low (HR = 0.83, 95% CI: 0.60–1.06; *p* = 0.12; Fig. 1G). Dichotomisation was performed at the cohort median (median = 1.78) using a univariate model. A complementary quartile-based stratification analysis similarly revealed no statistically significant association between AGR2 expression levels and overall survival (Supplementary Fig. 1B). Patients were stratified into four groups (Q1–Q4) according to AGR2 mRNA expression, with Q1 (lowest AGR2 expression) used as the reference category. Overall survival probabilities were estimated using the Kaplan–Meier method and compared using the log-rank test, which revealed no significant difference across the four quartiles (*p* = 0.31), with no individual comparison reaching statistical significance (all *p* > 0.05). Given the continuous, unimodal distribution of AGR2 expression (Fig. 1C and D), binary or quartile classification may not accurately reflect biologically distinct subtypes, which could explain the absence of significant survival differences between these groups. Taken together, these data demonstrate concordant transcriptomic and proteomic AGR2 expression and indicate that the heterogeneity observed by immunohistochemistry reflects a continuous gradient of molecular variation rather than distinct tumour subgroups.

**Table 1:** Immunohistochemical assessment of AGR2 expression in a local EOC cohort (n = 19).

Tumour	AGR2 IHC Score
NF184 (01B)	-
NG702 (01H*)	-
NH022 (01 N)	-
NJ013 (01H)	-
NJ229 (01E)	-
NJ464 (01S)	-
NM961 (02 K)	-
NN866 (11X)	-
NA183 (01 A)	+
NF815 (1TF)	+
NG174 (01 F)	+
NG395 (01 A)	+
NI504 (02 A)	+
NJ268 (01 J)	+
NK088 (01 A)	+
NN739 (02 A)	+
NO003 (02 C)	+
NG679 (01 F)	++
NH378 01	++

**Table 2:** Descriptive and distribution shape metrics of AGR2 expression in the TCGA-OV transcriptomic and TCGA-OV proteomic cohorts.

Parameters	TCGA_OV_proteomic	TCGA_OV_transcriptomic
AGR2 expression unit	(log₂ relative abundance / pooled reference)	(log₂ counts per million — log₂CPM)
Number of samples (n)	203	417
Mean expression	-0.414	1.939
Median expression	-0.489	1.781
Standard deviation (SD)	1.049	2.42
Largest gap (absolute)	0.5	0.82
Gap / Range ratio	0.08	0.058
Hartigan’s Dip (D)	0.017	0.015
Dip test p-value	0.9747	0.8288
Silverman’s test (p)	0.8643	0.9296
Gaussian Mixture Model (best components)	2	1
Bimodality Coefficient (BC)	0.29	0.166

### AGR2 expression is associated with matrisome and EMT

**Fig. 2:**
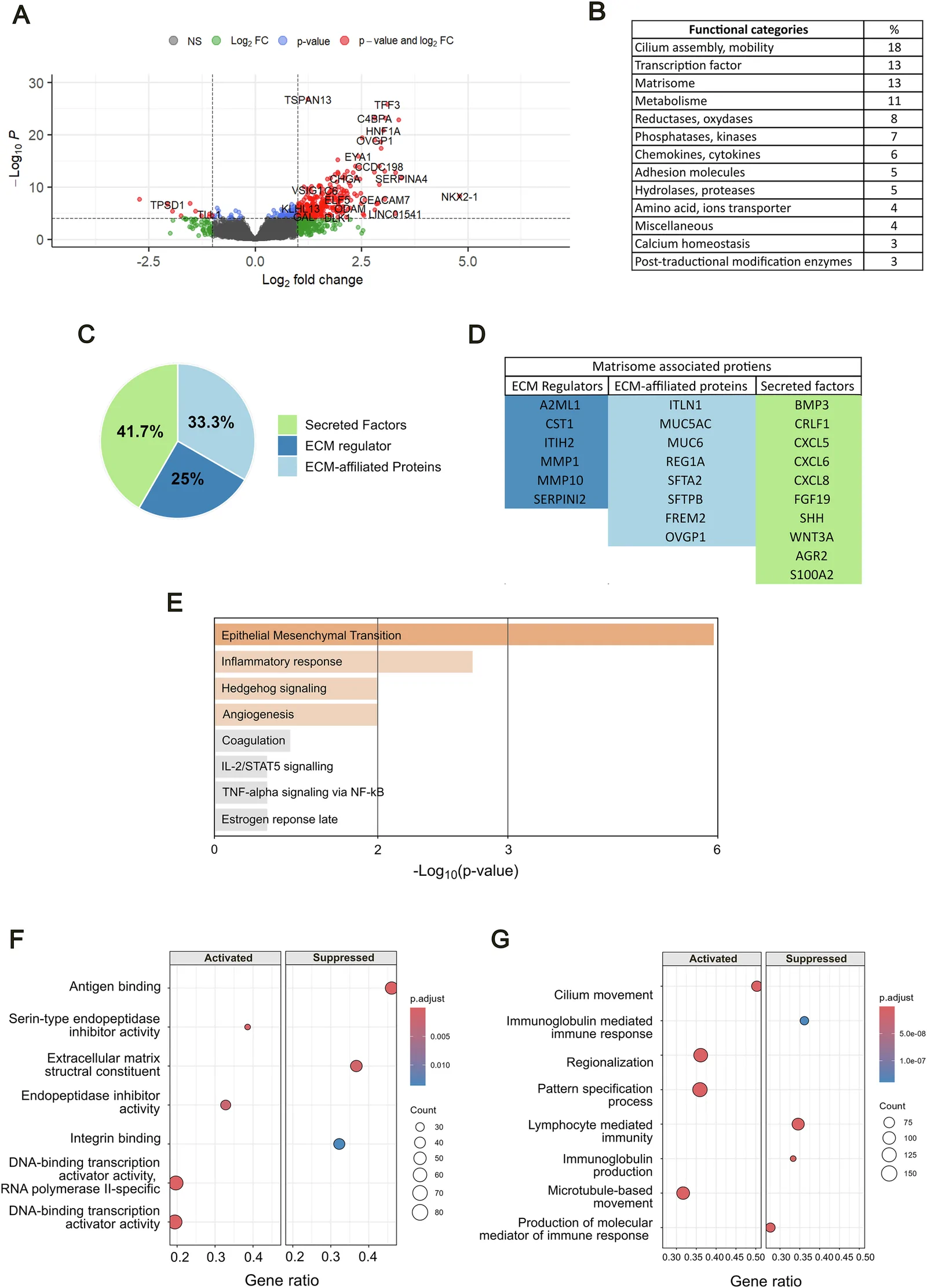
AGR2 expression correlates with matrisome and EMT. **A** Volcano plot representing differentially expressed genes (DEGs) in the TCGA-OV dataset between tumours with high versus low AGR2 mRNA content. The log10 (p-value) was plotted against log2 fold change values for all the significantly differentially expressed genes at 5% false discovery rate (FDR) with at least a two-fold change between both AGR2 groups. **B** Table showing gene classes expressed as a percentage of the total differentially expressed genes between high and low AGR2 groups in TCGA-OV data. **C** Pie chart showing the proportional distribution of matrisome-associated genes. The Matrisome classification was based on a validated Matrisome gene list [61]. **D** List of matrisome-associated proteins identified in our dataset, organized according to their functional categories: extracellular matrix (ECM) regulators, ECM-affiliated proteins, and secreted factors. **E** MSigDB-GSEA analysis of secreted factors associated with the matrisome revealed that the top enrichment score was obtained for epithelial-mesenchymal transition (EMT) pathways, with a significance level of *p* < 10⁻⁵. **F, G** Functional enrichment analysis of AGR2-associated differentially expressed genes (DEGs) using Gene Ontology (GO) categories. Significantly enriched terms are shown for activated (upregulated) and suppressed (downregulated) genes. Dot colour reflects adjusted p-values and dot size corresponds to the number of DEGs in each term.

Starting from the observation that AGR2 expression is upregulated and heterogeneous among ovarian tumours, we next investigated the biological processes associated with its expression in patient datasets extracted from the TCGA. Tumours were stratified into AGR2-high and AGR2-low groups based on median expression. Differential expression analysis identified distinct gene sets between the two groups, with the overall distribution illustrated by a volcano plot (Fig. 2A). Functional classification of genes upregulated in the AGR2-high subgroup highlighted the matrisome as one of the most prominent categories (Fig. 2B). Within this category, secreted factors accounted for 41.7% (n = 10) of differentially expressed genes, followed by ECM regulators (33.3%, n = 8) and ECM-affiliated proteins (25%, n = 6) (Fig. 2C, D). Given the central role of secreted factors in remodelling the tumour microenvironment, we next examined their contribution to AGR2-associated pathways. Gene set enrichment analysis (GSEA, MSigDB gene sets) revealed significant enrichment of epithelial-to-mesenchymal transition (EMT)-related pathways (Fig. 2E). As a first-pass analysis in TCGA-OV, median stratification and GSEA represent appropriate approaches to identify pathways associated with AGR2. In addition, GSEA indicated activation of collagen fibril organisation (p < 0.002) alongside with relative suppression of fibronectin binding and ECM adhesion (p < 0.002), suggesting a shift from stable to more dynamic ECM interactions (Fig. 2F, G). Collectively, these transcriptomic analyses indicate that tumours with high AGR2 expression are characterised by ECM remodelling and EMT activation, supporting a potential role for AGR2 in promoting migratory behaviour in EOC.

### Generation and characterisation of EOC cell lines overexpressing AGR2

**Fig. 3:**
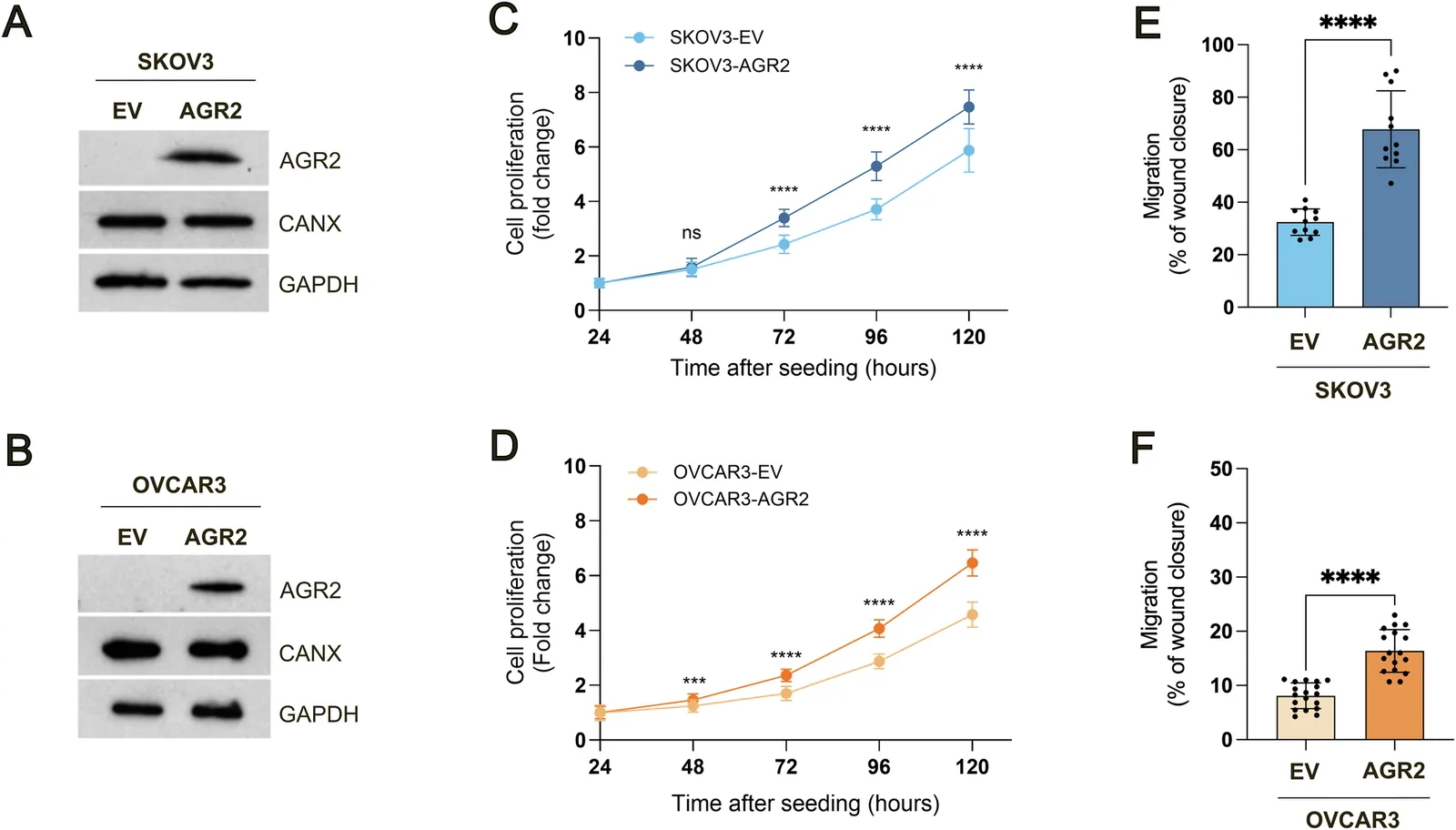
AGR2 confers tumour cell aggressiveness advantages to AGR2-negative cell lines. **A**, **B** Western Blot analysis showing AGR2 protein expression in AGR2-transduced cell lines compared with empty vector controls (EV). GAPDH served as loading control. One representative experiment (n = 3) is shown. **C**, **D** Cell proliferation was monitored over 120 h in SKOV3 (**C**) and OVCAR3 (**D**) models. Daily cell counts were performed, and cell numbers per field area were normalized to the 24 hour time point (t = 24 h). Data are mean ± SD from three independent experiments; p-values were calculated using a two-tailed unpaired Student’s *t*-test (*ns* = not significant, *** *p* < 0.001, ****, *p* < 0.0001). **E**, **F** Quantification of wound closure was performed based on image analysis over the 48-hour period in SKOV3-EV and SKOV-AGR2 (**E**) and OVCAR3-EV and OVAR3-AGR2 (**F**) models. Data are presented as mean ± SD from three independent experiments. Data are mean ± SD from three independent experiments; p-values were calculated using a two-tailed unpaired Student’s *t*-test (**** *p* < 0.0001).

To investigate the functional role of AGR2 in ovarian tumorigenesis, OVCAR3 and SKOV3 cell lines were selected due to their minimal endogenous AGR2 expression, providing a null background to specifically study the effects of intra- and extracellular AGR2 (Supplementary Fig. 2A). Considering the heterogeneous nature of EOC, which spans a spectrum of epithelial and mesenchymal phenotypes [40], we next assessed the epithelial–mesenchymal status of these cell lines. Transcriptomic profiling using a curated panel of 44 epithelial and 33 mesenchymal markers as defined by Kohn et al. [35], showed that OVCAR3 retained an epithelial-like gene expression profile, whereas SKOV3 exhibited a predominantly mesenchymal phenotype (Supplementary Fig. 2B). Based on these findings, OVCAR3 was selected as a representative model of high-grade serous ovarian cancer (HGSOC) with an epithelial phenotype, while SKOV3 served as a mesenchymal-like model. Employing these two cell lines allowed coverage of two distinct extremes within the EOC aggressiveness spectrum. Moreover, their minimal endogenous AGR2 expression established an optimal ‘null background’ for subsequent gain-of-function studies. Immunohistochemical (IHC) analysis further supported these classifications. SKOV3 cells, consistent with a mesenchymal profile, expressed vimentin but lacked E-cadherin, SOX2, and p16 (Supplementary Fig. 2C). In contrast, OVCAR3 cells displayed an epithelial profile, with E-cadherin expression, absence of vimentin, and detectable SOX2 and p16 (Supplementary Fig. 2C). p53 immunoreactivity also reflected their genetic backgrounds: SKOV3 (*TP53*-WT) showed no detectable protein, whereas OVCAR3 (*TP53*^R248Q^ mutant) displayed strong nuclear accumulation. Stable expression of AGR2 was achieved in both cell lines through lentiviral transduction. Immunofluorescence staining detected AGR2 in a high proportion of transduced cells (OVCAR3-AGR2 and SKOV3-AGR2) (Supplementary Fig. 3A, B), and quantitative image analysis estimated a transduction efficiency of approximately 80% (Supplementary Fig. 3C, D). AGR2 expression was further confirmed at the transcript level by RT-qPCR (Supplementary Fig. 3E, F) and at the protein level by western blotting (Fig. 3A, B). Together, these data confirm that OVCAR3-AGR2 and SKOV3-AGR2 cell lines actively express AGR2 and constitute suitable models for investigating its functional role in EOC.

To assess the functional impact of AGR2 overexpression in these cells, we evaluated cell proliferation and migratory capacity. Cell proliferation was monitored over a 120-hour time course, demonstrating that AGR2 overexpression conferred a 30% increase in proliferation in SKOV3-AGR2 cells compared with SKOV3-EV controls (Fig. 3C), and a 40% increase in OVCAR3-AGR2 cells compared with OVCAR3-EV controls (Fig. 3D). We next examined whether AGR2 overexpression influenced cell migration using wound-healing assays (Fig. 3E, F**;** Supplementary Fig. 3G, H). AGR2-expressing cells closed wounds significantly faster than their EV controls. In SKOV3 cells, migration increased from 32.4% for SKOV3-EV cells to 67.8% for SKOV3-AGR2 cells (Fig. 3E), whereas in OVCAR3 cells, migration increased from 8.1% for OVCAR3-EV cells to 16.4% for OVCAR3-AGR2 cells (Fig. 3F), corresponding to a two-fold increase in both models. As expected, OVCAR3-EV cells displayed lower overall migration capacity than SKOV3-EV cells, consistent with their more epithelial phenotype. Scratch closure reflects both migration and proliferation; no mitotic inhibitor was used in these assays. Altogether, AGR2 overexpression increased proliferation and accelerated scratch closure under serum-free conditions, consistent with enhanced motility, thereby supporting a functional role for AGR2 in tumour development.

### Extracellular eAGR2 enhances tumorigenic process in EOC cells

**Fig. 4:**
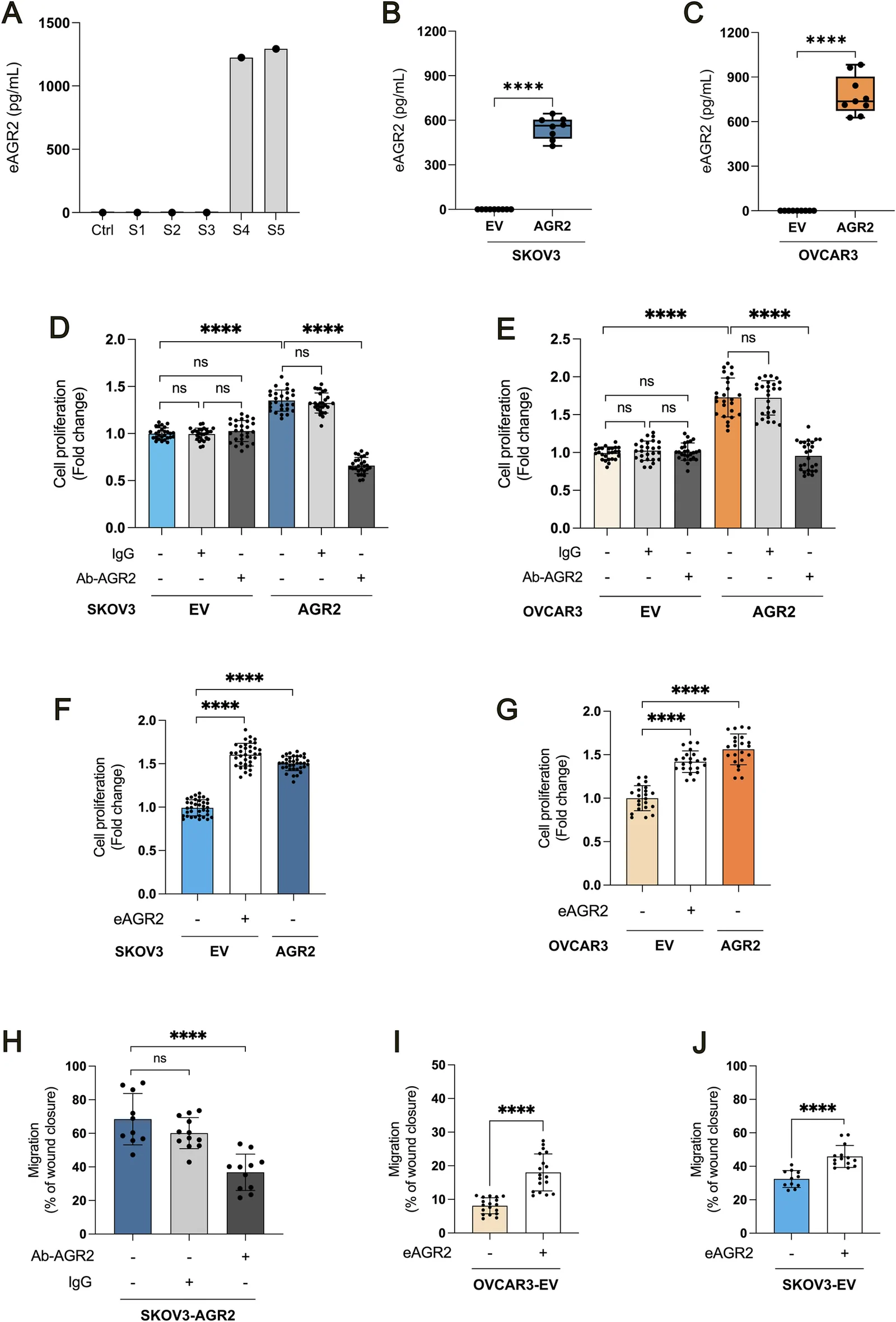
eAGR2 enhances tumour cell aggressiveness in EOC Cells. **A–C** The concentration of extracellular AGR2 (eAGR2) in the culture supernatants of epithelial ovarian cancer PDTOs (n = 5) (**A**), SKOV3-EV and SKOV-AGR2 (**B**) and OVCAR3-EV and OVCAR3-AGR2 (**C**) cells was measured using the Gyrolab® automated immunoassay system. **B, C** Data are mean ± SD from three independent experiments; p-values were calculated using a two-tailed unpaired Student’s *t*-test (**** *p* < 0.0001). **D, E** Effect of an AGR2-neutralising antibody (Ab-AGR2; 40 µg/mL) on cell proliferation in SKOV3 (**D**) and OVCAR3 (**E**) cells. Cells transfected with either EV or AGR2-overexpressing vectors were treated with a non-relevant isotype control (IgG) or Ab-AGR2. Proliferation was measured after 72 h and is expressed as fold change relative to the corresponding EV control for each cell line. Data represent mean ± SD from three independent experiments. P-values were determined by a two-tailed unpaired Student’s t-test (ns = not significant, **** *p* < 0.0001). **F,** **G** Effect of extracellular recombinant AGR2 (200 ng/mL) on cell growth in SKOV3-EV (**F**) and OVCAR3-EV (**G**) cells after 72 hours. Data are mean ± SD from three independent experiments; p-values were calculated using a two-tailed unpaired Student’s *t*-test (**** *p* < 0.0001). **H**–**J** Quantification of wound closure was performed based on image analysis over the 48-hour period in SKOV3-AGR2 (**H**), OVCAR3-EV (**I**) and SKOV3-EV (**J**). Data are mean ± SD from three independent experiments; *p*-values were calculated using a two-tailed unpaired Student’s *t*-test (**** *p* < 0.0001).

We next hypothesised that extracellular AGR2 (eAGR2) might contribute to cell proliferation and migration in EOC. To test this, we first measured eAGR2 secretion in the supernatants (S1–S5) of five EOC Patient-Derived Tumour Organoids (PDTO) (Table 3). Quantitative immunoassays revealed marked heterogeneity, with some tumoroids actively secreting high levels of eAGR2, while others displayed little or no secretion (Fig. 4A). As shown in Table 3, the PDTO models used in this study represent two major histological subtypes of epithelial ovarian cancer: High-Grade Serous Carcinoma (HGSC; models OV-057 and OV-015) and Clear Cell Carcinoma (CCC; model OV-009). Interestingly, the highest levels of eAGR2 secretion (Fig. 4A) were observed in the CCC model (OV-009) and one of the HGSC models (OV-015), suggesting that eAGR2 release may vary depending on the specific tumour background. This variability highlights inter-patient differences in eAGR2 secretion, consistent with the heterogeneous AGR2 expression pattern observed in our local ovarian tumour cohort (Fig. 1B). To further expand these data, EOC cell lines were examined, and we confirmed by quantitative immunoassays that both OVCAR3-AGR2 and SKOV3-AGR2 cell lines secrete eAGR2 (Fig. 4B, C). To evaluate the functional impact of eAGR2 on proliferation, SKOV3-AGR2 and OVCAR3-AGR2 cells were treated with a neutralising anti-AGR2 antibody. Blocking eAGR2 (Ab-AGR2) significantly reduced cell proliferation in both models, with approximately 50% reduction in OVCAR3-AGR2 cells and 40% in SKOV3-AGR2 cells (Fig. 4D, E). As expected, a non-relevant IgG control antibody had no effect on the proliferation of OVCAR3-EV and SKOV3-EV control cells (Fig. 4D, E), confirming that the observed inhibition was specific to eAGR2 neutralisation. Furthermore, the use of a non-relevant isotype control, together with the lack of effect in AGR2-negative cell lines, supports the specificity of the antibody under our experimental conditions. Conversely, addition of recombinant AGR2 protein in the culture medium of AGR2-negative SKOV3-EV and OVCAR3-EV cells restored cell proliferation capacity to levels comparable with AGR2-expressing SKOV3-AGR2 and OVCAR3-AGR2 cells (Fig. 4F, G). To ensure a saturating signal and optimal reproducibility across all functional readouts, these experiments were conducted using 200 ng/mL of recombinant AGR2. To confirm the biological relevance of this dosage, we performed a dose–response curve (0–400 ng/mL) across three cell models (SKOV3, OVCAR3, and A549shAGR2). These results (Supplementary Fig. 4A–C) demonstrate a dose-dependent pro-proliferative effect in all lines, with statistical significance reached from 40 ng/mL. These observations are in full agreement with our previous reports [18, 25] and confirm that AGR2 exerts its pro-tumoural functions at concentrations close to physiological levels. Under these experimental conditions (200 ng/mL), proliferation increased by 1.4-fold and 1.5-fold in OVCAR3-AGR2 and SKOV3-AGR2 cells, respectively, relative to their EV counterparts, and increased 1.5-fold and 1.6-fold in OVCAR3-EV and SKOV3-EV cells, respectively, following treatment with recombinant AGR2 protein. To assess effects on migration, neutralisation of eAGR2 with an anti-AGR2 antibody in SKOV3-AGR2 cells reduced wound closure by nearly 50% (from 68.4% to 36.8%), indicating that eAGR2 promotes cell motility (Fig. 4H, Supplementary Fig. 4D). Consistently, addition of recombinant AGR2 enhanced migration in both cell models (Fig. 4I, J**;** Supplementary Fig. 4E, F). In OVCAR3 cells, migration increased more than 2-fold (from 8.1% to 18.0%) (Fig. 4I), whereas in SKOV3 cells, it increased 1.4-fold (from 32.4% to 45.9%) (Fig. 4J). To determine whether the effect of eAGR2 on cell migration was associated with the acquisition of a mesenchymal phenotype, IHC analyses were performed on SKOV3 and OVCAR3 cells cultured in the presence or absence of recombinant AGR2 (eAGR2). In both cell lines, AGR2 treatment significantly reduced E-cadherin expression (Supplementary Fig. 2D, E). In contrast, neither vimentin (Supplementary Fig. 2F, G) nor SOX2 expression (Supplementary Fig. 2H, I) was significantly altered in the presence of eAGR2. These findings suggest that the pro-migratory effect of eAGR2 is associated with a reduction in epithelial characteristics rather than with a complete mesenchymal transition. Taken together, these assays demonstrate that eAGR2 is sufficient to stimulate EOC cell proliferation and migration in vitro; antibody neutralisation is consistent with necessity under these conditions.

**Table 3:** Evaluation of eAGR2 in Patient-Derived Tumour Organoid (PDTO).

Sample	Description	Subtype	Study_NO	Lvalue	Units	%cv
Ctrl	Organoid medium	N/A	EE-0312e	< 9.77	pg/mL	N/A
S1	Organoid OV-057_T2	HGSC	EE-0312e	< 9.77	pg/mL	N/A
S2	Organoid OV-057_T1	HGSC	EE-0312e	< 9.77	pg/mL	N/A
S3	Organoid OV-057_A	HGSC	EE-0312e	< 9.77	pg/mL	N/A
S4	Organoid OV-009_T	CCC	EE-0312e	1224.80	pg/mL	2.05
S5	Organoid OV-015_A	HGSC	EE-0312e	1293.06	pg/mL	0.17

*HGSC* High Grade Serous Carcinoma, *CCC*, Clear Cell Carcinoma, *N/A*, Not Available.

### Characterisation of the eAGR2-induced functional network

**Fig. 5:**
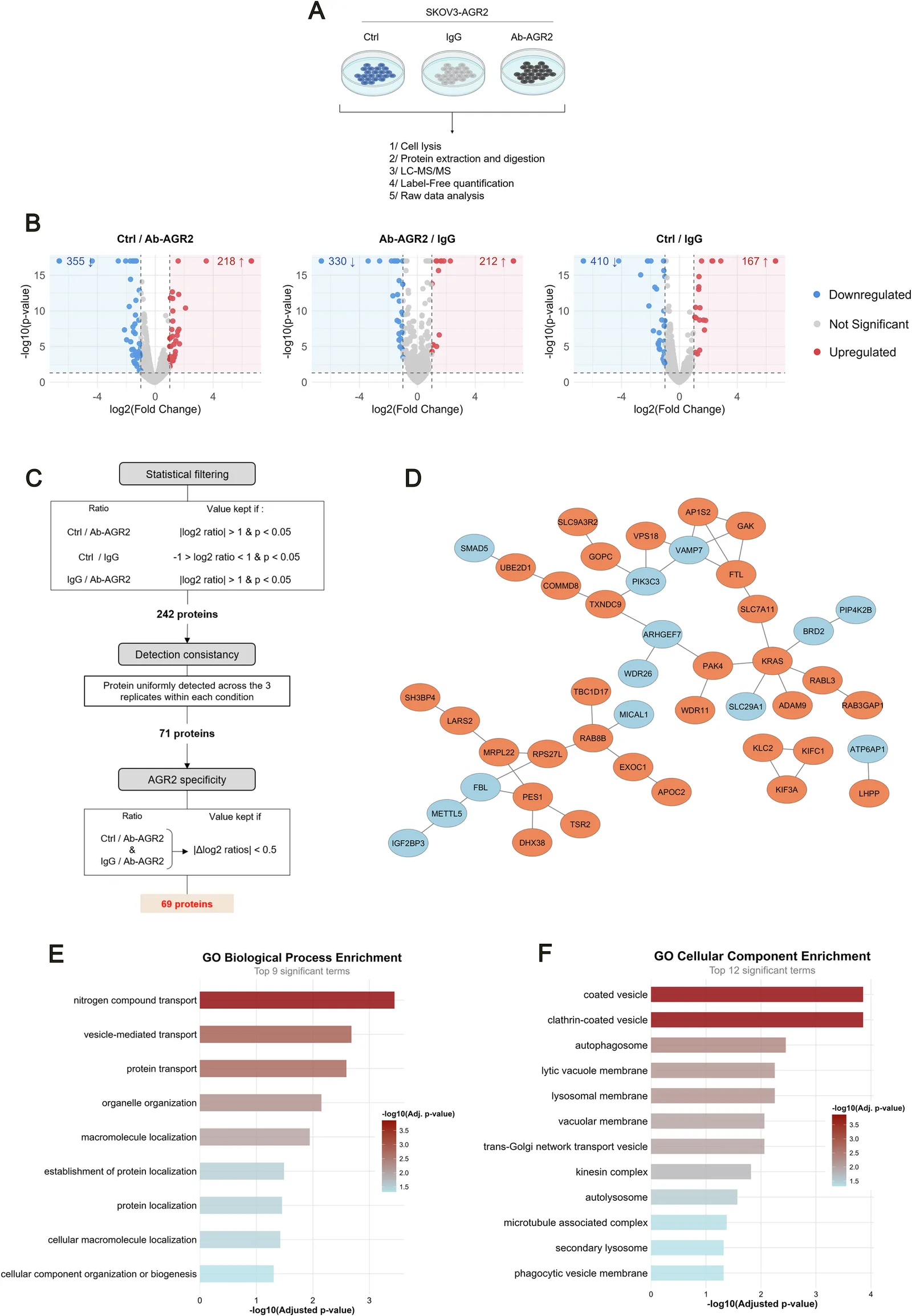
Proteomic workflow and integrative analysis identifying proteins modulated by eAGR2. **A** Schematic overview of the proteomic workflow used to investigate the impact of extracellular AGR2 (eAGR2) on the intracellular tumour proteome. SKOV3-AGR2 cells were treated for 72 h with an AGR2-neutralizing antibody (Ab-AGR2) or an IgG non-relevant isotype antibody (IgG). Following treatment, cells were lysed, proteins were extracted and digested. Proteomic analysis was performed by label-free LC-MS/MS, and data were processed using Proteome Discoverer 3.1. **B** Volcano plots showing quantitative proteomic comparisons between the experimental conditions. Each dot represents a protein quantified by label-free LC–MS/MS analysis. Significantly upregulated and downregulated proteins are shown in red and green, respectively ( | log₂FC | > 1; *p* < 0.05). Comparisons correspond to Ctrl vs. Ab-AGR2 (left), Ab-AGR2 vs. IgG (middle), and Ctrl vs. IgG (right). The number of proteins meeting these criteria is indicated in each quadrant. **C** Data filtering strategy for proteomic analysis. Differentially abundant proteins were successively filtered based on statistical significance, detection consistency across replicates, and AGR2 specificity, yielding a final list of 69 candidate proteins specifically modulated by eAGR2. **D** Cytoscape network visualization generated from the curated list of proteins identified by the proteomic analysis. Each node represents a protein, and edges indicate functional associations. Orange nodes correspond to upregulated proteins, while blue nodes represent downregulated proteins. This network illustrates the functional relationships between proteins modulated in response to extracellular AGR2 (eAGR2). **E,** **F** Gene Ontology (GO) enrichment analysis of the curated protein list identified from the proteomic dataset. Enrichment analyses were performed separately for Biological Process (**E**) and Cellular Component (**F**) categories using over-representation analysis. Only significantly enriched GO terms (adjusted *p*-value < 0.05) are displayed. Bars represent the most significant terms ranked by log₁₀ (adjusted p-value), illustrating the main biological processes (**E**) and cellular component (**F**) associated with the proteins modulated in response to extracellular AGR2 (eAGR2).

Next, we aimed to characterise the intracellular proteomic alterations induced by eAGR2. To this end, we used our SKOV3-AGR2 model which exhibited the strongest eAGR2-mediated effects on cell proliferation and migration (Fig. 4D, H), and compared proteomic profiles under the three following conditions (Fig. 5A): (i) untreated SKOV3-AGR2 cells (Ctrl), (ii) SKOV3-AGR2 cells treated with a non-relevant IgG antibody (IgG), and (iii) SKOV3-AGR2 cells treated with a neutralising anti-AGR2 antibody. Prior to mass spectrometry analysis, we confirmed that anti-AGR2 antibody treatment effectively reduced proliferation compared with IgG and Ctrl conditions, validating the functional blockade of eAGR2 (Supplementary Fig. 5A). Cell lysates from each condition were analysed by label-free LC-MS/MS proteomics, and quantitative comparisons were performed across the three conditions (Fig. 5B). To identify proteins specifically modulated by eAGR2, three pairwise comparisons were performed: Ctrl vs. Ab-AGR2, IgG vs. Ab-AGR2, and Ctrl vs. IgG. Abundance ratios (fold-change, FC) and associated *p*-values were calculated for each comparison. Proteins were considered significantly regulated in the Ctrl vs. Ab-AGR2 and Ab-AGR2 vs. IgG comparisons when |log₂FC | > 1 and *p* < 0.05. To exclude non-specific IgG effects, only proteins showing minimal variation (–1 < log₂FC < 1) or not detected (NA) in the Ctrl vs. IgG comparison were retained. Moreover, only proteins consistently detected across all replicates were included to ensure robustness. Proteins with an absolute difference (Δlog₂FC) < 0.5 were retained, ensuring specificity to AGR2 inhibition. Finally, this filtering reduced the dataset from 568 to 69 proteins (12.1% of the initial identifications) (Fig. 5C), which were considered eAGR2-modulated candidates (Supplementary Fig. 5B). To explore the functional relationships among eAGR2-modulated proteins, a functional interaction network analysis was conducted using Cytoscape. The resulting network comprised 44 nodes and 47 edges, with a clustering coefficient of 0.169 and a density of 0.101, revealing four interconnected clusters **(**Fig. 5D). This organisation is consistent with eAGR2 influencing groups of functionally related proteins and is hypothesis-generating. Enrichment analysis of biological processes (GO:BP) indicated that eAGR2 blockade is associated with changes in intracellular trafficking pathways, including nitrogen compound transport (*p* = 3.58 × 10⁻⁴), vesicle-mediated transport (*p* = 2.07 × 10⁻³), and protein transport (*p* = 2.55 × 10⁻³) (Fig. 5E, Supplementary Fig. 5C). These processes collectively encompass the biosynthetic and degradative flux of macromolecules through the endomembrane system, are consistent with eAGR2-associated changes in trafficking between the endoplasmic reticulum, Golgi apparatus, and lysosomal compartments. Such regulation supports endocytosis–exocytosis cycles and proper protein localisation, processes essential for phagosome formation, maturation, and intracellular trafficking. Further subcellular context analysis (GO:CC) revealed that the most affected compartments were vesicle-associated structures, notably clathrin-coated vesicles (*p* = 1.39 × 10⁻⁴) and trans-Golgi network transport vesicles (*p* = 8.66 × 10⁻³), highlighting active cargo sorting and Golgi–plasma membrane communication (Fig. 5F, Supplementary Fig. 5D). Enrichment in autophagosomes (*p* = 3.54 × 10⁻³), lysosomal membranes (*p* = 5.61 × 10⁻³), and autolysosomes (*p* = 2.68 × 10⁻²) further supports a role for eAGR2 in modulating the degradative branch of vesicular transport, particularly the autophagy–phagosome–lysosome axis. Consistent with these enriched categories, several regulators of vesicular trafficking and autophagy were differentially modulated in SKOV3-AGR2 cells treated with a neutralising anti-AGR2 antibody, including upregulation of RAB8B, VPS18, TBC1D17, and SH3BP4, and downregulation of PIK3C3 (VPS34) and VAMP7 (Table 4). Together, these findings suggest that eAGR2 may modulate multiple components of the autophagic and transport machinery.

**Table 4:** eAGR2-modulated proteins involved in endocytic and autophagic membrane trafficking.

Protein	Family name	Biological process	Cellular Component	Protein Class
PIK3C3	Phosphatidylinositol 3-kinase family (Class III)	Autophagosome assembly	Peroxisome, Endosome, Phagophore assembly site	Kinase
RAB8B	RAB small GTPase family	Exocytosis, Protein secretion, Endocytic recycling	Endosome, Trans-Golgi network transport vesicle, Plasma membrane	Small GTPase
SH3BP4	SH3 domain–binding protein family	Regulation of endocytosis	Cytoplasm, Endosome, Plasma membrane	Scaffold/adaptor protein
TBC1D17	TBC1 domain family member 17	Regulation of endocytosis, control of Rab8/Rab11 recycling pathways, autophagy regulation	Cytosol, Endosome, Membrane-associated	GTPase-activating protein
VAMP7	Vesicle-associated membrane protein 7	Vesicle fusion, endosome to lysosome transport, exocytosis	Vesicle membrane, Lysosome, Endosome	SNARE protein
VPS18	Vacuolar protein sorting-associated protein 18 homologs	Endosome–lysosome fusion, protein sorting to lysosome, autophagy	Membrane protein complex, Endosome, Vesicle tethering complex	Membrane trafficking regulatory protein

### eAGR2 enhances global protein synthesis in tumour cells

**Fig. 6:**
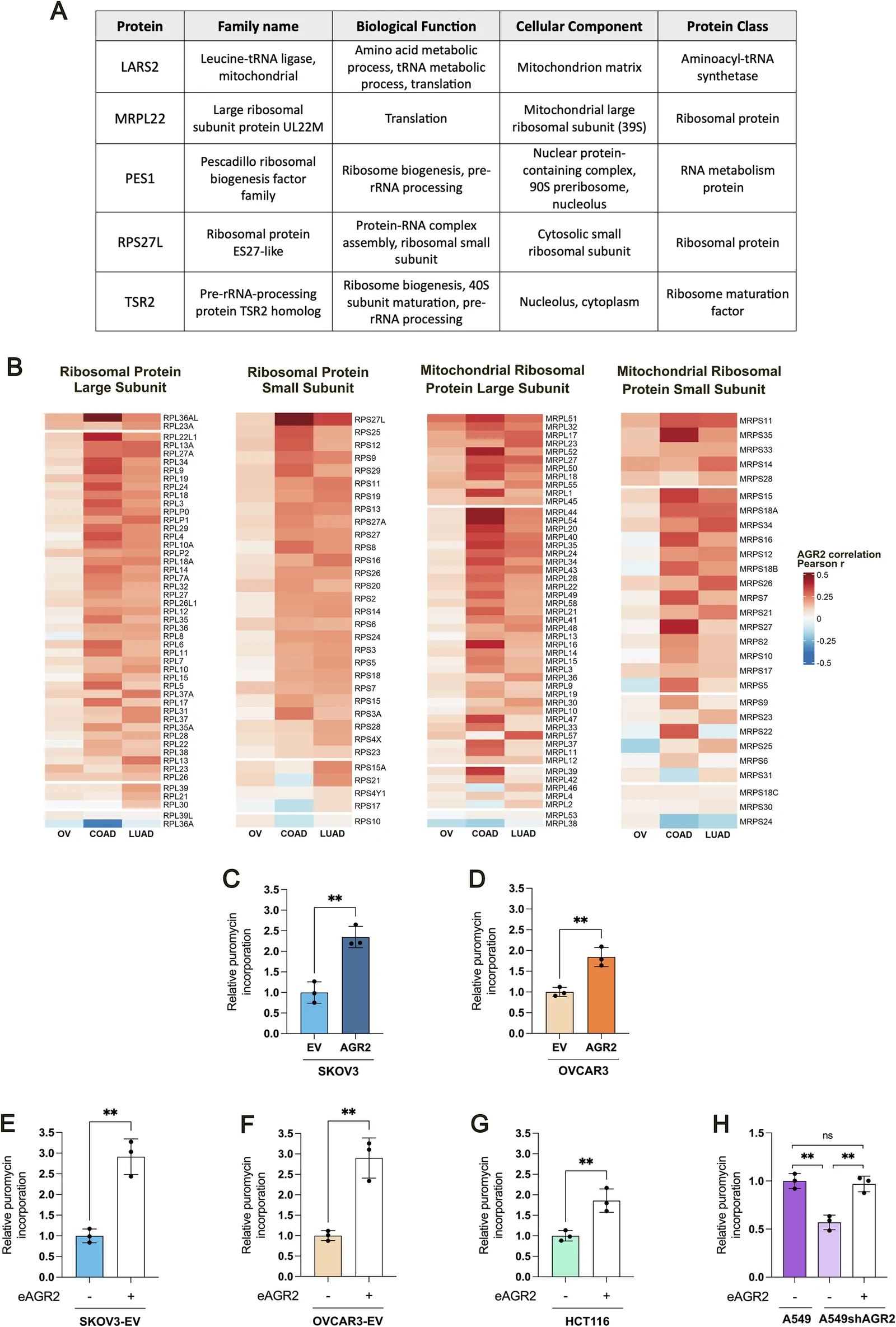
Extracellular AGR2 modulates ribosome-associated proteins and enhances global protein synthesis across adenocarcinomas. **A** The table summarizes the family name, main biological function, subcellular localisation, and protein class of five proteins identified in our proteomic dataset as significantly modulated by eAGR2 and functionally associated with ribosome biogenesis or translation. **B** Heatmap displaying Pearson correlation coefficients (r) between AGR2 and ribosomal protein genes (cytosolic RPL/RPS and mitochondrial MRPL/MRPS) across the three TCGA RNA-seq cohorts: Ovarian Cancer (OV), Colon adenocarcinomas (COAD) and Lung Adenocarcinomas (LUAD). Positive correlations are shown in red and negative correlations in blue. **C–H** Puromycin incorporation was quantified using ImageJ by integrating the total lane intensity of the SUnSET smear. For each condition, values were normalized to calnexin (loading control), and equal loading was verified by Ponceau Red staining (Supplementary Figure 4). **C**, **D** show SKOV3 and OVCAR3 cells stably expressing AGR2 compared with empty vector (EV) controls. **E**, **G** correspond to SKOV3, OVCAR3, and HCT116 cells stimulated with recombinant AGR2 (200 ng/mL) versus untreated controls for 72 h (**H**) A549 and A549 sh-AGR2 cells cultured in the presence or absence of recombinant AGR2 for 72 h. In all cases, increased puromycin signal reflects enhanced global protein synthesis in response to AGR2 expression or recombinant AGR2 stimulation. Data represent mean ± SD from three independent experiments, and statistical comparisons were performed using a two-tailed unpaired Student’s t-test (**p < 0.01).

Given that eAGR2 signalling modulates components of the autophagic machinery, we hypothesized that this function might support intracellular amino acid availability and, consequently, enhance protein synthesis, thereby maintaining proteostasis. In our network analysis, we identified a subset of interconnected proteins involved in translation and ribosome biogenesis that were upregulated under active eAGR2 signalling (Fig. 5D, Fig. 6A). These included MRPL22 and LARS2, components of the mitochondrial ribosome, RPS27L, a cytosolic ribosomal protein, and PES1 and TSR2, factors involved in ribosome biogenesis and maturation. In contrast, FBL, a nucleolar methyltransferase essential for rRNA processing, was downregulated, suggesting that eAGR2 may differentially modulate specific steps of ribosome assembly. To determine whether this observation reflected a broader association between AGR2 and ribosomal pathways across adenocarcinomas, we examined AGR2 mRNA correlations with ribosome-related genes, including cytoplasmic (RPL, RPS) and mitochondrial (MRPL, MRPS) ribosomal proteins, using TCGA datasets from ovarian (OV), colon (COAD), and lung (LUAD) adenocarcinomas (Fig. 6B). COAD and LUAD were selected as additional adenocarcinomas, as we and others have demonstrated strong AGR2 expression and secretion in these tumour types [8, 18, 25, 26, 41–43]. This analysis revealed widespread positive correlations between *AGR2* and ribosomal gene expression, with many associations reaching high significance. The trend was consistent across all ribosomal families, encompassing both cytoplasmic (*RPL*, *RPS*) and mitochondrial (*MRPL*, *MRPS*) subunits, and was conserved across ovarian (OV), colon (COAD), and lung (LUAD) adenocarcinomas cohorts, appearing particularly strong in COAD and LUAD (Fig. 6B). To investigate the functional consequences of this association, we performed a puromycin-based SUnSET assay to measure global protein synthesis (Fig. 6C – H, Supplementary Fig. 6B, C). In both OVCAR3 and SKOV3 models, cells stably overexpressing AGR2 (OVCAR3-AGR2 and SKOV3-AGR2) exhibited a marked increase in puromycin incorporation compared with their respective controls (OVCAR3-EV and SKOV3-EV), indicating enhanced translational activity upon AGR2 overexpression (Fig. 6C, D). To confirm the contribution of eAGR2, AGR2-negative cell lines (OVCAR3-EV and SKOV3-EV) were treated with recombinant AGR2 protein (Fig. 6E, F). In both cases, eAGR2 treatment increased puromycin-labelled nascent peptides, demonstrating that eAGR2 directly promotes protein synthesis. A similar effect was observed in the HCT116 colorectal cancer cell line (Fig. 6G), chosen as an AGR2-negative colorectal model (Suppl. Fig. 6A). Conversely, in the AGR2-positive A549 lung cancer cell line, silencing AGR2 expression led to a marked decrease in protein synthesis, while the addition of recombinant AGR2 to silenced cells restored protein synthesis levels (Fig. 6H, Suppl. Fig. 6D). Collectively, these findings demonstrate that AGR2 expression is consistently associated with ribosomal and ribosome-related genes across multiple adenocarcinomas, and establish that eAGR2 can functionally enhance global protein synthesis in tumour cells. Collectively, these findings demonstrate that AGR2 expression is consistently associated with ribosomal and ribosome-related genes across multiple adenocarcinomas and establish that extracellular AGR2 can functionally enhance overall production of proteins in tumour cells.

### eAGR2 modulates the expression autophagy-related proteins and sustains translational activity under autophagic stress

**Fig. 7:**
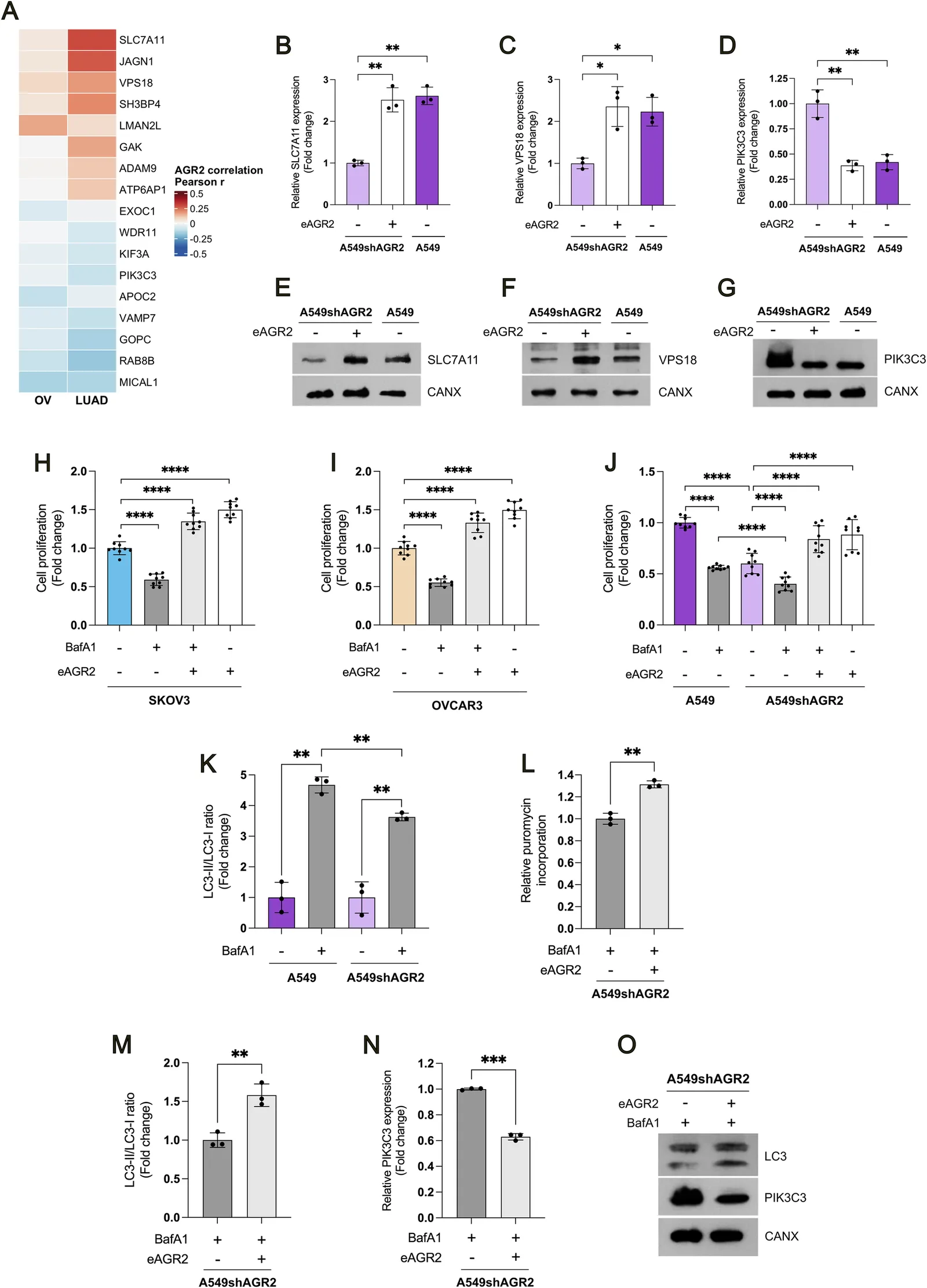
Extracellular AGR2 modulates autophagy and vesicle transport pathways to promote cell proliferation and protein synthesis. **A** Heatmap displaying Pearson correlation coefficients between AGR2 mRNA expression and the expression of selected eAGR2-modulated proteins identified in the proteomic dataset across TCGA ovarian (OV) and lung adenocarcinoma (LUAD) cohorts. Positive correlations are shown in red and negative correlations in blue. **B–D** Quantification of relative protein expression by Western Blot analysis for SLC7A11 (**B**), VPS18 (**C**), and PIK3C3 (**D**) in A549shAGR2 cells treated with recombinant AGR2 (200 ng/mL) compared with untreated A549shAGR2 and parental A549 controls. Values were normalised to calnexin (CANX) and expressed as fold change relative to the A549shAGR2 untreated condition. Data are mean ± SD from three independent experiments; *P*-values were calculated using a two-tailed unpaired Student’s t-test (*p < 0.05, **p < 0.01). **E–G** Representative Western Blot images corresponding to the quantifications shown in (**B–D**). **H**, **I** Effect of Bafilomycin A1 (BafA1; 150 nM) treatment and/or recombinant AGR2 stimulation (200 ng/mL) on cell proliferation in SKOV3 (**H**) and OVCAR3 (**I**) cells. Cell proliferation was expressed as fold change relative to the untreated control condition of each respective cell line. Data are mean ± SD from three independent experiments; *P*-values were calculated using a two-tailed unpaired Student’s t-test (****p < 0.0001). **J** Effect of BafA1 treatment (150 nM) and/or recombinant AGR2 stimulation (200 ng/mL) on cell proliferation in parental A549 and AGR2-silenced A549shAGR2 cells. Cell proliferation across all conditions was expressed as fold change relative to the untreated parental A549 control. Data are mean ± SD from three independent experiments; *P*-values were calculated using a two-tailed unpaired Student’s t-test (****p < 0.0001). **K** LC3-II/LC3-I ratio assessed by Western Blot in A549 and A549shAGR2 cells in the presence or absence of BafA1 (150 nM). Values were normalised to CANX and expressed as fold change relative to the untreated control of each respective cell line. Data are mean ± SD from three independent experiments; *P*-values were calculated using a two-tailed unpaired Student’s t-test (**p < 0.01). **L** Global protein synthesis assessed by puromycin incorporation (SUnSET assay) in A549shAGR2 cells treated with BafA1 (150 nM) in the presence or absence of recombinant AGR2 (200 ng/mL). Values are expressed as fold change relative to the BafA1-treated, eAGR2-untreated condition. Data are mean ± SD from three independent experiments; p-values were calculated using a two-tailed unpaired Student’s t-test (**p < 0.01). **M** Quantification of LC3-II/LC3-I ratio in A549shAGR2 cells treated with BafA1 (150 nM) in the presence or absence of recombinant AGR2 (200 ng/mL). Values were normalised to CANX and expressed as fold change relative to the BafA1-treated, eAGR2-untreated condition. Data are mean ± SD from three independent experiments; *p*-values were calculated using a two-tailed unpaired Student’s t-test (**p < 0.01). **N** Quantification of PIK3C3 expression in A549shAGR2 cells treated with BafA1 (150 nM) in the presence or absence of recombinant AGR2 (200 ng/mL). Values were normalised to CANX and expressed as fold change relative to the BafA1-treated, eAGR2-untreated condition. Data are mean ± SD from three independent experiments; *P*-values were calculated using a two-tailed unpaired Student’s t-test (***p < 0.001). **O** Representative Western Blot analysis of LC3, PIK3C3, and CANX in A549shAGR2 cells treated with BafA1 (150 nM) in the presence or absence of recombinant AGR2 (200 ng/mL), corresponding to the quantifications shown in (**M**) and (**N**). CANX served as a loading control.

Having established that eAGR2 enhances global protein synthesis, we next sought to validate whether the autophagy-, vesicle transport-, and lysosome-related proteins identified in our proteomic interaction network (Fig. 5D) correlated at the transcriptomic level with AGR2 expression in patient cohorts. Pearson correlation analysis was performed across TCGA ovarian (OV) and lung adenocarcinoma (LUAD) cohorts for proteins identified in these key functional pathways. We found that a significant subset of these candidates displayed transcriptomic profiles globally mirroring the proteomic modulation observed in our network (Fig. 7A). Specifically, proteins whose expression was upregulated upon active eAGR2 signalling, such as SLC7A11, VPS18, GAK, ADAM9, and ATP6AP1, showed positive mRNA correlations with *AGR2*, whereas downregulated proteins, including PIK3C3, VAMP7, and MICAL1, showed negative correlations, with consistent patterns across both tumour types (Fig. 7A). Among these concordant candidates, *SLC7A11*, *VPS18*, and *PIK3C3* were selected for protein-level validation given their established roles in autophagy initiation, vesicular trafficking, and lysosomal function. Western Blot analyses in A549shAGR2 cells treated with recombinant AGR2 confirmed that eAGR2 stimulation significantly increased SLC7A11 and VPS18 protein levels compared with untreated A549shAGR2 controls, restoring expression towards levels observed in parental A549 cells (Fig. 7B, C, E, F; Suppl, Fig. 7A, B). Conversely, PIK3C3 protein levels were significantly reduced upon recombinant AGR2 treatment, recapitulating the expression pattern observed in AGR2-expressing parental A549 cells (Fig. 7D, G; Suppl. Fig. 7C). To further explore the functional relationship between eAGR2 and autophagy, we assessed cell proliferation under pharmacological autophagy blockade using Bafilomycin A1 (BafA1). BafA1 treatment significantly reduced cell proliferation in SKOV3 and OVCAR3 cells, while the addition of recombinant AGR2 significantly rescued this phenotype (Fig. 7H, I). Consistently, BafA1 treatment reduced proliferation in both A549 and A549shAGR2 cells; however, the inhibitory effect was more pronounced in A549shAGR2 cells than in parental A549 cells (Fig. 7J). Addition of recombinant AGR2 to BafA1-treated A549shAGR2 cells partially rescued proliferation, further confirming the capacity of eAGR2 to counteract proliferative inhibition induced by autophagy blockade (Fig. 7J). To assess whether the differential proliferative response between A549 and A549shAGR2 cells under BafA1 treatment was associated with differences in autophagic flux, LC3-II/LC3-I ratios were compared. BafA1 treatment significantly increased the LC3-II/LC3-I ratio in both cell lines, confirming effective autophagy blockade (Fig. 7K; Suppl. Fig. 7D, E). Notably, the magnitude of LC3-II accumulation was greater in parental A549 cells than in A549shAGR2 cells, suggesting that AGR2 expression is associated with higher basal autophagic flux, consistent with the attenuated proliferative response to BafA1 observed in this model (Fig. 7J, K). Given the proliferation rescue observed upon eAGR2 stimulation in BafA1-treated A549shAGR2 cells, we next characterised the molecular changes accompanying this response. Global protein synthesis, assessed by puromycin incorporation, was significantly increased in BafA1-treated A549shAGR2 cells treated with recombinant AGR2 compared with BafA1 treatment alone (Fig. 7L; Suppl. Fig. 7F). Alongside this increase in translational activity, the LC3-II/LC3-I ratio was further elevated in recombinant AGR2-stimulated cells compared with BafA1 treatment alone (Fig. 7M, N; Suppl. Fig. 7F). Recombinant AGR2 stimulation also significantly reduced PIK3C3 protein levels under BafA1 treatment (Fig. 7N, O; Suppl. Fig. 7F), consistent with the downregulation observed in untreated A549shAGR2 cells. Taken together, these results confirm the signaling networks identified at both transcript and proteins levels. Furthermore, they functionally establish a link between eAGR2, autophagy, and protein synthesis, demonstrating that eAGR2 modulates key autophagy- and vesicular trafficking regulators to sustain translational activity even under conditions of autophagic stress.

## Discussion

In this study, we show that eAGR2 promotes malignant features in EOC together with iAGR2. Indeed, our data support a synergistic model in which AGR2 operates across two compartments. Extracellular eAGR2 appears to promote global protein synthesis not through canonical growth signalling, but by coordinating metabolic and translational processes, while iAGR2 safeguards protein folding in the ER. By coupling translational expansion with proteostasis, AGR2 contributes to establishing a self-reinforcing loop that sustains the elevated biosynthetic demands of tumour cells. This integrative framework provides a unifying view of AGR2 function in cancer. We show that eAGR2 promotes proliferation and migration in EOC cells, consistent with its activity across multiple adenocarcinomas. In breast [44], pancreatic [31] and lung [26] cancer, eAGR2 sustains tumour proliferation. Its pro-migratory role is also conserved: in prostate [45], lung [46], and colorectal [47] cancer, eAGR2 enhances invasion, in part by inducing EMT. Our findings indicate that the pro-migratory effect of eAGR2 in ovarian cancer cell models is associated with a reduction in epithelial characteristics, reminiscent of a partial EMT state, rather than a complete mesenchymal transition. In pancreatic ductal adenocarcinoma (PDAC), tumour-promoting signalling is amplified by a reciprocal paracrine AGR2/IGF1 loop between cancer cells and cancer-associated fibroblasts, driving desmoplasia and tumour progression [48]. Furthermore, eAGR2 contributes to immune evasion in LKB1-deficient tumours by promoting dendritic cell dysfunction and impairing the efficacy of anti-PD-1 immunotherapy, particularly in lung adenocarcinoma models [49]. Our transcriptomic analysis of the TCGA ovarian cohort further links high AGR2 expression with matrisome and EMT programs. Functionally, our cell models recapitulate these observations, as neutralisation of eAGR2 reduced migration while recombinant AGR2 restored motility. Together, these findings reinforce the view that eAGR2 contributes to matrix remodelling and invasive behaviour.

While these results highlight the role of AGR2 in remodelling the tumour microenvironment, this study focused on characterising the novel mechanisms by which eAGR2 coordinates intracellular autophagy and translation to meet the high biosynthetic requirements of ovarian cancer cells. Beyond this conserved tumour-promoting role, our findings reveal that eAGR2 modulates components of intracellular trafficking and autophagy, positioning it as a regulator of metabolic homeostasis in tumour cells. Consistently, the enrichment of vesicle-mediated transport and lysosomal compartments observed in our proteomic analysis highlights a role for eAGR2 in maintaining the autophagy–lysosome axis. Examination of individual proteins modulated under active eAGR2 signalling further supports this view. Notably, RAB8B and VPS18 were modulated, both involved in vesicle trafficking and autophagosome–lysosome fusion processes essential for cargo degradation and recycling [50–52]. This link is further supported by our transcriptomic analysis of TCGA cohorts, where AGR2 expression positively correlates with SLC7A11 and VPS18, while showing a negative correlation with PIK3C3. We confirmed these findings at the protein level, demonstrating that eAGR2 stimulation restores SLC7A11 and VPS18 expression while downregulating PIK3C3 in A549shAGR2 cells, mirroring the parental cell phenotype. The regulation of SLC7A11 is particularly significant, as it mediates cystine import, which is essential for glutathione synthesis and survival under oxidative stress. By simultaneously coordinating SLC7A11 expression and autophagy, eAGR2 likely ensures both amino acid supply for translation and protection against ferroptosis, reinforcing its role as a global protector of tumour survival in hostile microenvironments.

Modulation of TBC1D17 and SH3BP4 further supports a link between vesicle dynamics and nutrient sensing: TBC1D17 acts as a GAP for Rab5 and Rab7, coordinating endocytic and mitophagic transport [53], while SH3BP4 modulates amino acid–dependent mTORC1 activation via Rag GTPases, linking nutrient signalling to autophagic control [54]. Moreover, eAGR2 modulates PIK3C3 (VPS34) and VAMP7, which are required for autophagosome initiation and vesicle fusion [55–57]. In addition to supporting autophagic cargo recycling and nutrient availability, AGR2 provides a further metabolic advantage by suppressing ferroptosis via the p53/FPN1 regulatory axis, thereby protecting cancer cells from iron-dependent oxidative stress [58]. Functionally, we demonstrated that eAGR2 significantly rescues the proliferative inhibition induced by the autophagy blocker Bafilomycin A1 (BafA1) across multiple cell models. This rescue capacity suggests that eAGR2 acts as a metabolic rheostat, maintaining a sufficient autophagic flux to fuel protein synthesis without triggering the excessive stress that leads to cell death. Together, these observations suggest that eAGR2 contributes to maintaining a balanced autophagic flux that supports vesicular turnover and nutrient availability while preventing excessive autophagy, thereby facilitating sustained protein synthesis. This protective mechanism closely aligns with the consequences observed upon iAGR2 depletion. Importantly, this model is consistent with our previous study (JBC 2011), in which iAGR2 depletion increased autophagic flux and sensitised cells to ER stress-induced autophagy, thereby linking intracellular AGR2 loss to proteostatic failure. Together, these observations support a model in which intracellular and extracellular AGR2 pools are functionally coordinated with the autophagic machinery to maintain proteostasis and cellular fitness, rather than acting as independent effectors. Further mechanistic work will be required to define causality.

Our proteomic analyses indicate the modulation of several components associated with autophagy and lysosomal pathways, together with the overall increase in protein synthesis observed through SUnSET analyses. This supports a functional hypothesis in which eAGR2 may promote translational activity by influencing the autophagic–lysosomal system and regulating intracellular amino-acid availability.

Given that autophagy plays a central role in maintaining intracellular amino acid availability, we hypothesised that eAGR2 may enhance the intracellular amino acid pool by promoting controlled autophagic activity, thereby supporting increased protein synthesis. In support of this hypothesis, our network analysis identified an interconnected cluster of ribosome-associated proteins upregulated under active eAGR2 signalling. This set included components of both mitochondrial and cytosolic translation machinery (MRPL22, LARS2, and RPS27L) as well as factors involved in ribosome biogenesis (PES1 and TSR2). The coordinated induction of these proteins is consistent with a global enhancement of translational capacity, coupling metabolic supply with biosynthetic demand. Extending this observation to patient datasets, we found that AGR2 transcriptional expression positively correlates with ribosomal and ribosome-associated genes across ovarian, colon, and lung adenocarcinomas, suggesting a conserved link between AGR2 and translational machinery. Functionally, stimulation of AGR2-negative cells with recombinant AGR2 increases puromycin incorporation, consistent with enhanced global translation. Moreover, we established that eAGR2 sustains this translational activity even under conditions of autophagic blockade, as evidenced by increased puromycin incorporation in BafA1-treated cells following eAGR2 addition. The fact that eAGR2 can bypass lysosomal inhibition may imply potential involvement in resistance to therapies targeting autophagic or metabolic pathways, as it provides a compensatory mechanism to sustain protein synthesis. Together, these findings suggest that eAGR2 contributes to the coordination of metabolic and translational pathways to sustain the biosynthetic activity of tumour cells, rather than definitively demonstrating a causal mechanism. Such fine-tuning of vesicle trafficking, autophagy, and translation is particularly relevant in cancer cells, which operate under chronic metabolic stress and rely on tightly regulated degradative and biosynthetic programmes to sustain tumour cell aggressiveness.

As a member of the protein disulphide isomerase (PDI) family, iAGR2 catalyses disulphide bond formation and isomerisation, ensuring the correct folding of secretory and transmembrane proteins [16]. Cancer cells, faced with high metabolic and proliferative demands, frequently experience ER stress and rely on the UPR to maintain proteostasis [59]. AGR2 is ER stress-inducible and contributes directly to this adaptive response. A particularly relevant example is its interaction with mucins, which are heavily glycosylated, cysteine-rich proteins strongly upregulated in many cancers [60], where they promote tumour growth and dissemination. This interplay between translational expansion and ER folding highlights the coordinated roles of iAGR2 and eAGR2 in sustaining tumour development. Before folding can occur, the cell must expand its translational capacity to ensure proper maturation of complex proteins. Consistent with this, our data suggest that eAGR2 contributes to maintaining metabolic activity by modulating vesicular trafficking and the autophagosome–lysosome axis, thereby potentially facilitating access to the resources required for increased translation. Direct demonstration of this mechanism would require further functional analyses of autophagic flux and intracellular amino acid availability. In line with this, our comparison of LC3-II/LC3-I ratios under BafA1 treatment revealed that eAGR2 is associated with significantly higher autophagic flux, providing a functional link between extracellular AGR2 and the degradative capacity of the cell.

The mechanistic basis underlying the effects of eAGR2 remains incompletely understood. At the cell surface, eAGR2 may interact with membrane partners such as the GPI-anchored protein LYPD3/C4.4 A [60], known to promote tumour progression, cell adhesion, and migration. Such interactions could influence signalling pathways controlling endocytic uptake and membrane trafficking, consistent with our observation of increased endocytosis. In line with our previous demonstration of eAGR2 localisation at the plasma membrane [26], these findings support a non-cell-autonomous role. eAGR2 could therefore be internalised via receptor-mediated endocytosis and subsequently access intracellular compartments. We speculate that this process may contribute to a feedback mechanism whereby eAGR2 enhances endocytic activity, thereby facilitating its own uptake. Following internalisation, eAGR2 may intersect with endosomal–lysosomal pathways or nutrient-sensing networks, potentially modulating autophagic flux. However, the efficiency of uptake, intracellular trafficking routes, and functional consequences of eAGR2, whether mediated by extracellular signalling, internalisation, or both, remain to be experimentally defined.

**Fig. 8:**
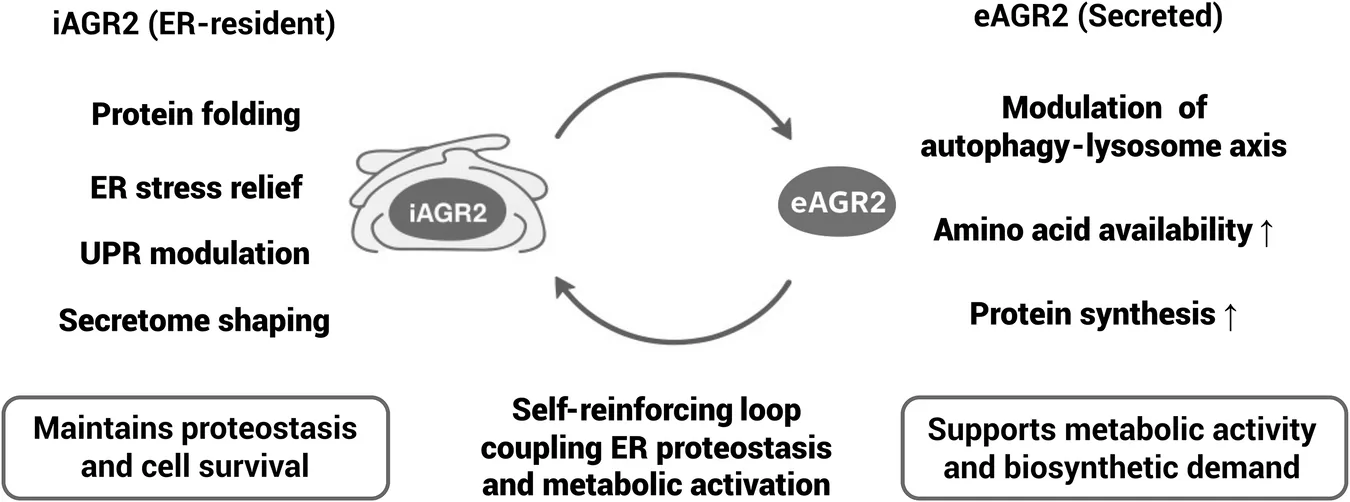
Dual-compartment model of AGR2 coupling ER proteostasis (iAGR2) and extracellular metabolic activation (eAGR2) in a self-reinforcing loop that enhances tumour cell aggressiveness. Intracellular AGR2 (iAGR2), functions as an ER-resident PDI that promotes protein folding, catalyses disulphide bond formation, alleviates ER stress, and regulates the unfolded protein response (UPR), thereby maintaining proteostasis and supporting cell survival under stress. Meanwhile, extracellular AGR2 (eAGR2) modulates the autophagy–lysosome axis, enhancing amino acid recycling and sustaining global protein synthesis. By linking intracellular folding capacity with extracellular metabolic and translational control, iAGR2 and eAGR2 establish a self-reinforcing loop that coordinates protein homeostasis and translation. Collectively, this dual-compartment regulation supports the high biosynthetic activity characteristic of tumour cells and drives tumour cell aggressiveness, integrating ER homeostasis, vesicular trafficking, and metabolic activation into a unified regulatory model.

Together, iAGR2 and eAGR2 appear to sustain a functional loop in which extracellular signalling and intracellular folding are coupled. eAGR2 may stimulate translation and metabolic fluxes, while iAGR2 enhances proteostasis, jointly supporting high protein synthesis and tumour development. A schematic representation of this dual-compartment regulatory loop is provided in Fig. 8. Antibody-mediated inhibition of eAGR2 disrupts this coordination, reducing autophagic activity and nutrient supply, imposing a metabolic constraint, and ultimately decreasing protein synthesis and tumour cell aggressiveness. Overall, our data support a model in which eAGR2 alleviates a metabolic constraint on cancer cell aggressiveness via effects on autophagic and lysosomal pathways. Neutralisation of eAGR2 disrupts this regulatory loop, limiting translational capacity and restraining tumour cell aggressiveness, highlighting eAGR2 as a potential coordinator linking nutrient homeostasis to tumour development rather than a sole causal driver. A potential limitation of this study is the inherent heterogeneity of EOC. We addressed this by using OVCAR3 and SKOV3 as complementary models of aggressive EOC phenotypes. While these lines allowed for a precise dissection of AGR2-mediated signalling due to their low basal expression, our findings were further validated in PDTOs, large clinical datasets (TCGA) and other epithelial cancers, including colorectal and lung cancers. Importantly, we observed a positive correlation between AGR2 mRNA and protein levels in patient tumours. Although overall survival did not reach statistical significance using a median binary threshold (*p* = 0.14), our bioinformatics analysis nevertheless linked AGR2 to major signalling pathways such as EMT and the matrisome, suggesting that AGR2 levels reflect biological aggressiveness even when clinical mortality is influenced by other confounding factors. Indeed, a key finding of our study is that the AGR2-proteostasis axis acts as a fundamental mechanism that transcends the clinical diversity of epithelial cancers. This conserved feature across ovarian, colon, and lung adenocarcinomas provides a molecular rationale for the poor outcomes associated with high AGR2 expression. Nevertheless, we explicitly acknowledge that our current study primarily focuses on the most aggressive spectra of EOC (HGSC-like and mesenchymal-like). Given the distinct biological drivers of EOC, future studies including a broader range of less frequent subtypes, such as mucinous, low-grade serous, and clear cell carcinoma models, would be necessary to fully generalise our conclusions across the entire EOC landscape.

In conclusion, by coupling translational expansion with ER folding capacity, AGR2 may orchestrate a self-reinforcing loop that sustains tumour biosynthetic activity and progression. This dual-compartment model highlights AGR2 as both a metabolic coordinator (eAGR2) and a proteostasis actor (iAGR2). Ultimately, the identification of eAGR2 as a functional driver of tumour aggressiveness, despite the influence of external clinical variables on overall survival, emphasises its extracellular localisation as a potential therapeutic target. Targeting this secreted pool represents a pragmatic clinical strategy to disrupt the biosynthetic machinery across a broad spectrum of aggressive epithelial tumours, offering an accessible extracellular vulnerability regardless of their specific clinical subtype.

## Supplementary information

Supplemental material

## Data Availability

There are no restrictions on data and materials accessibility. The mass spectrometry proteomics data have been deposited to the Center for Computational Mass Spectrometry repository (University of California, San Diego) via the MassIVE tool with the dataset identifier MassIVE MSV000099646.
