# Supplementary material for: Complementary intra- and extracellular AGR2 activities support epithelial ovarian cancer cell aggressiveness
Source: Oncogene. 2026 Aug 10;45(40):4215–35. doi: 10.1038/s41388-026-03938-y (PMC13600914; doi:10.1038/s41388-026-03938-y)
Supplement: Supplementary file 1 [file 41388_2026_3938_MOESM1_ESM.pdf]

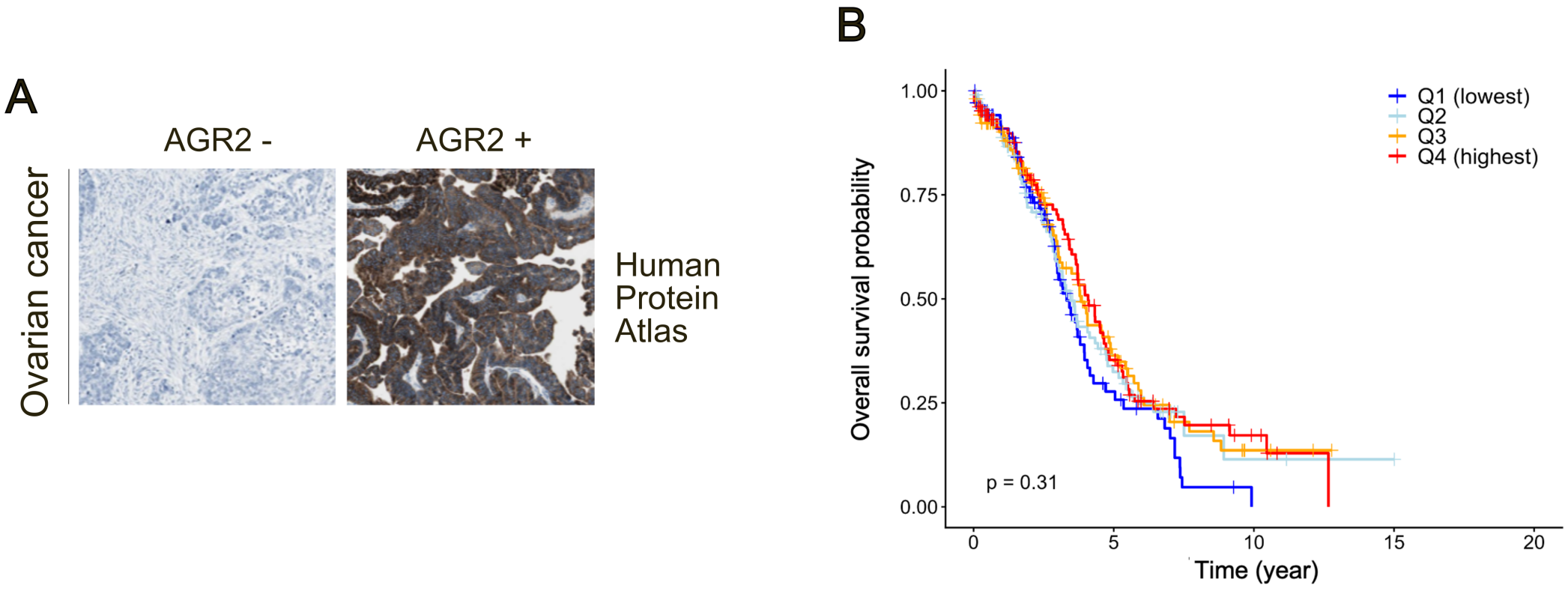

Supplemental Figure 1 – Histological AGR2 expression and quartile-based overall survival analysis in EOC patients.

(**A**) Representative immunohistochemical staining of AGR2 in ovarian cancer specimens from the Human Protein Atlas, illustrating AGR2-low (left) and AGR2-high (right) expression. (**B**) Kaplan–Meier analysis of overall survival in EOC patients from the TCGA-OV cohort (n = 415). Patients were stratified into four groups (Q1–Q4) according to AGR2 mRNA expression, with Q1 (lowest AGR2 expression) used as the reference category. Overall survival probabilities were compared across all four groups using the log-rank test (p = 0.31).

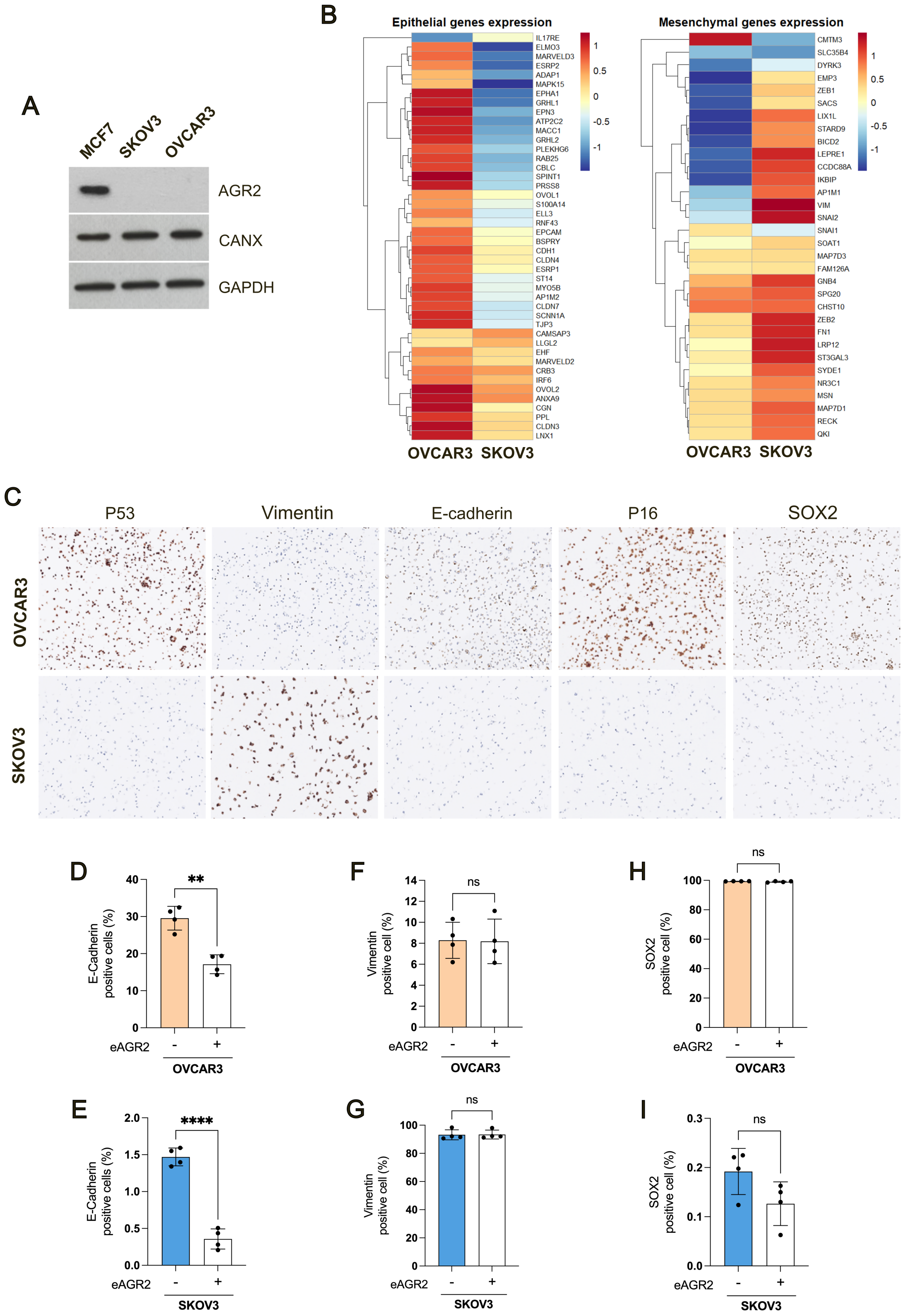
A
MCF7
SKOV3
OVCAR3
AGR2
CANX
GAPDH
B
Epithelial genes expression
IL17RE
ELMO3
MARVELD3
ESRP2
ADAP1
MAPK15
EPHA1
GRHL1
EPN3
ATP2C2
MACC1
GRHL2
PLEKHG6
RAB25
CBLC
SPINT1
PRSS8
OVOL1
S100A14
ELL3
RNF43
EPCAM
BSPRY
CDH1
CLDN4
ESRP1
ST14
MYO5B
AP1M2
CLDN7
SCNN1A
TJP3
CAMSAP3
LLGL2
EHF
MARVELD2
CRB3
IRF6
OVOL2
ANXA9
CGN
PPL
CLDN3
LNX1
OVCAR3 SKOV3
Mesenchymal genes expression
CMTM3
SLC35B4
DYRK3
EMP3
ZEB1
SACS
LIX1L
STARD9
BICD2
LEPRE1
CCDC88A
IKBIP
AP1M1
VIM
SNAI2
SNAI1
SOAT1
MAP7D3
FAM126A
GNB4
SPG20
CHST10
ZEB2
FN1
LRP12
ST3GAL3
SYDE1
NR3C1
MSN
MAP7D1
RECK
QKI
OVCAR3 SKOV3
C
P53
Vimentin
E-cadherin
P16
SOX2
OVCAR3
SKOV3
D
E-Cadherin positive cells (%)
**
eAGR2
OVCAR3
E
E-Cadherin positive cells (%)
****
eAGR2
SKOV3
F
Vimentin positive cell (%)
ns
eAGR2
OVCAR3
G
Vimentin positive cell (%)
ns
eAGR2
SKOV3
H
SOX2 positive cell (%)
ns
eAGR2
OVCAR3
I
SOX2 positive cell (%)
ns
eAGR2
SKOV3

Supplemental Figure 2 - Ovarian cancer cell line model characterization.

(**A**) Cell lysates from MCF7, SKOV3 and OVCAR3 were analysed by Western Blot using an anti-AGR2 antibody. MCF7 cells served as a positive control. GAPDH and CANX were used as loading controls. One representative experiment (n = 3) is shown. (**B**) Heatmap displaying the expression of 44 epithelial-associated genes and 33 mesenchymal-associated genes in SKOV3 and OVCAR3 cells. Gene selection was based on the curated EMT panel defined by Kohn *et al.* (36). Expression data were retrieved from the Cancer Cell Line Encyclopaedia (CCLE), $\log_2$-transformed, and z-score normalised across samples to enable direct comparison of epithelial and mesenchymal phenotypes. **(C)** Expression of p53, Vimentin, E-cadherin, p16, SOX2, and determined by immunohistochemistry in paraffin-embedded cell pellets from OVCAR3 and SKOV3 (×10 magnification). (**D–I**) Quantitative IHC analysis of (**D, E**) E-cadherin, (**F, G**) vimentin, and (**H, I**) SOX2 expression in OVCAR3 and SKOV3 cell pellets treated with or without recombinant AGR2 (eAGR2). Data are presented as the percentage of DAB-positive cells relative to the total cell count per region of interest (ROI). Results represent the mean ± SD of four independent experiments. P-values were determined using a two-tailed unpaired Student's t-test (ns = not significant, $^{**}p < 0.01$, and $^{***}p < 0.001$).

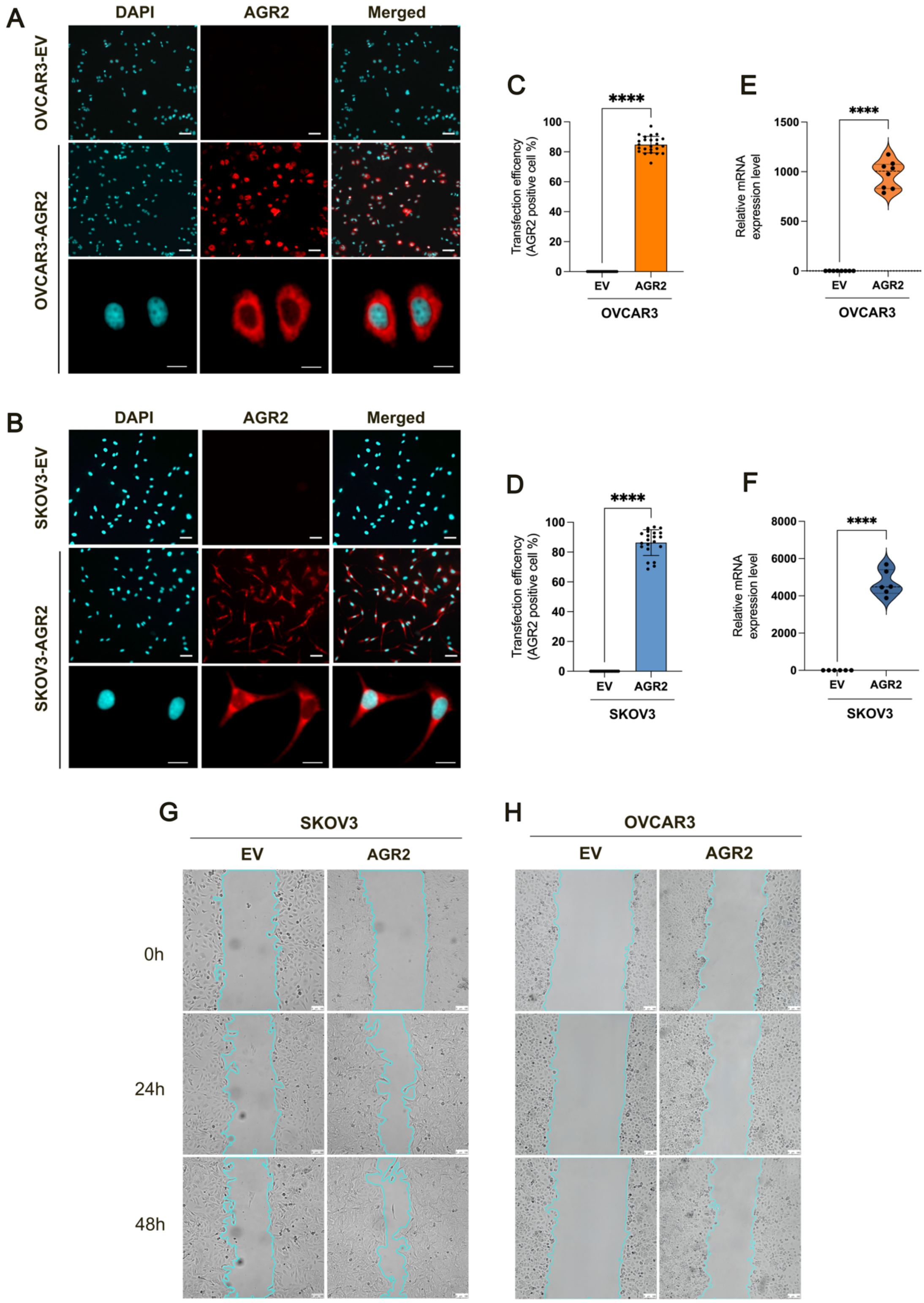
A
DAPI
AGR2
Merged
OVCAR3-EV
OVCAR3-AGR2
B
DAPI
AGR2
Merged
SKOV3-EV
SKOV3-AGR2
C
****
Transfection efficency (AGR2 positive cell %)
100
80
60
40
20
0
EV
AGR2
OVCAR3
E
****
Relative mRNA expression level
1500
1000
500
0
EV
AGR2
OVCAR3
D
****
Transfection efficency (AGR2 positive cell %)
100
80
60
40
20
0
EV
AGR2
SKOV3
F
****
Relative mRNA expression level
8000
6000
4000
2000
0
EV
AGR2
SKOV3
G
SKOV3
EV
AGR2
0h
24h
48h
H
OVCAR3
EV
AGR2

Supplemental Figure 3 - Validation of AGR2 overexpression and functional assays in OVCAR3 and SKOV3 models.

(**A, B**) Immunofluorescence analysis of AGR2 expression in OVCAR3 (**A**) and SKOV3 (**B**), Scale bars = 75 µm. (**C, D**) Quantification of transduction efficiency was performed by calculating the percentage of AGR2-positive cells relative to the total cell population (DAPI-stained nuclei), based on fluorescence signal intensity. Data are mean ± SD from three independent experiments; p-values were calculated using a two-tailed unpaired Student's *t*-test (**** $p < 0.0001$). (**E, F**) AGR2 expression was analysed using RT-qPCR and normalized to the mean expression of *GAPDH*. Data are mean ± SD from three independent experiments; p-values were calculated using a two-tailed unpaired Student's *t*-test (**** $p < 0.0001$). (**G, H**) Representative images of wound closure at 0 h (time of scratch), 24 h, and 48 h. Scale bar = 100 µm.

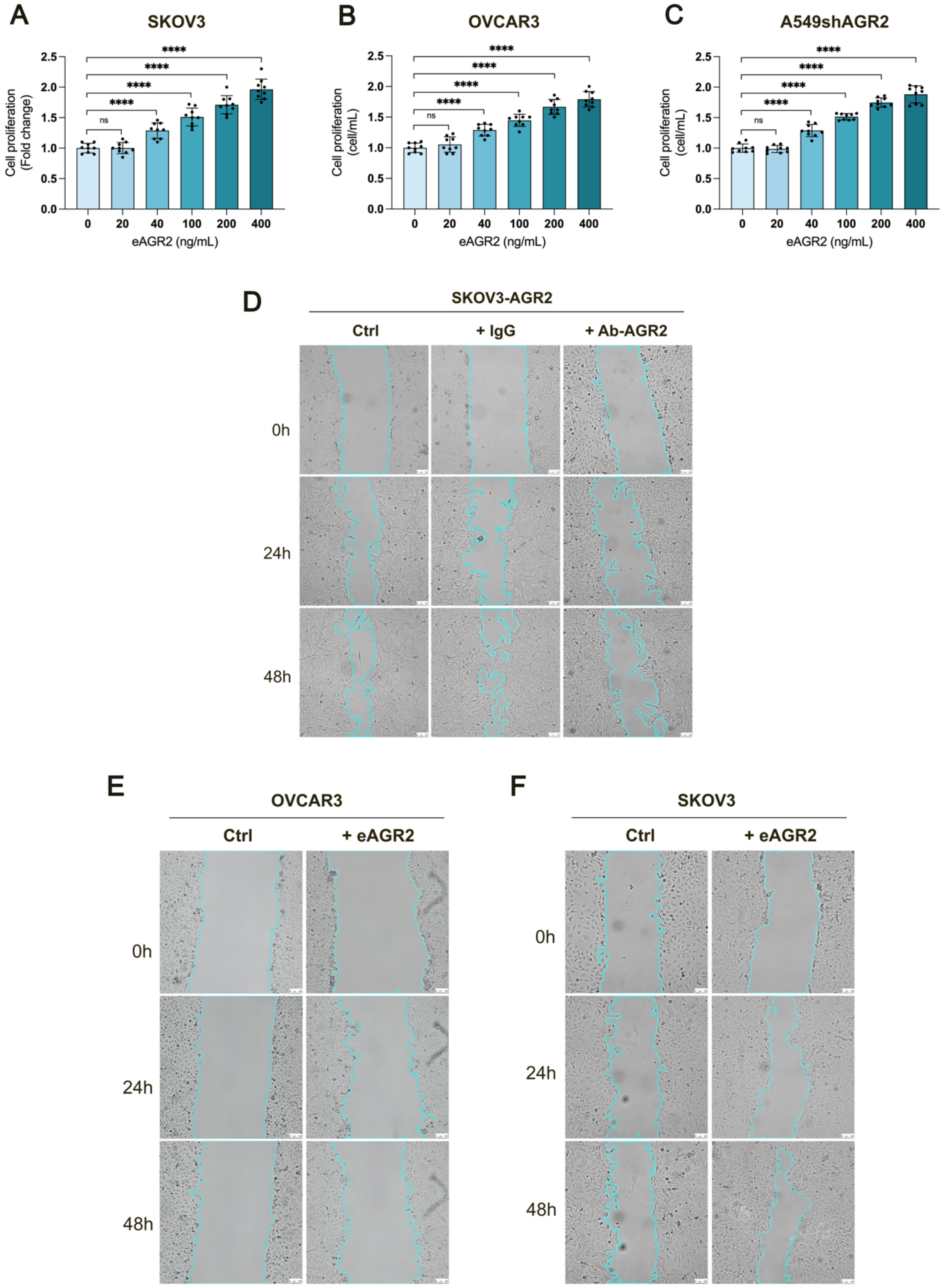
A
SKOV3
Cell proliferation (Fold change)
eAGR2 (ng/mL)
B
OVCAR3
Cell proliferation (cell/mL)
C
A549shAGR2
D
SKOV3-AGR2
Ctrl
+ IgG
+ Ab-AGR2
0h
24h
48h
E
OVCAR3
+ eAGR2
F
SKOV3

Supplemental Figure 4 - Effect of eAGR2 inhibition and recombinant protein treatment on SKOV3 and OVCAR3 cells.

(**A - C**) Dose-response effect of recombinant AGR2 on cell proliferation in SKOV3 (**A**), OVCAR3 (**B**), and A549shAGR2 (**C**) cell lines. Cells were treated with increasing concentrations of recombinant AGR2 (0, 20, 40, 100, 200, and 400 ng/mL). Cell proliferation was measured after 72 h by propidium iodide staining and expressed as fold change relative to the untreated control. Data are presented as mean ± SD from three independent experiments; p-values were calculated using a two-tailed unpaired Student's t-test (*ns* = not significant, **** $p < 0.0001$). (**D**) Representative images of wound closure at 0 h (time of scratch), 24 h, and 48 h following SKOV3-AGR2 treatment with either an AGR2 blocking antibody or a non-relevant isotype control antibody (both at 40 µg/mL) in SKOV3 cell. Scale bar = 100 µm. (**E, F**) Representative images of wound closure at 0 h (time of scratch), 24 h, and 48 h in SKOV3 (**E**) and OVCAR3 (**F**) cultured in the presence or absence of recombinant AGR2. Scale bar = 100 µm.

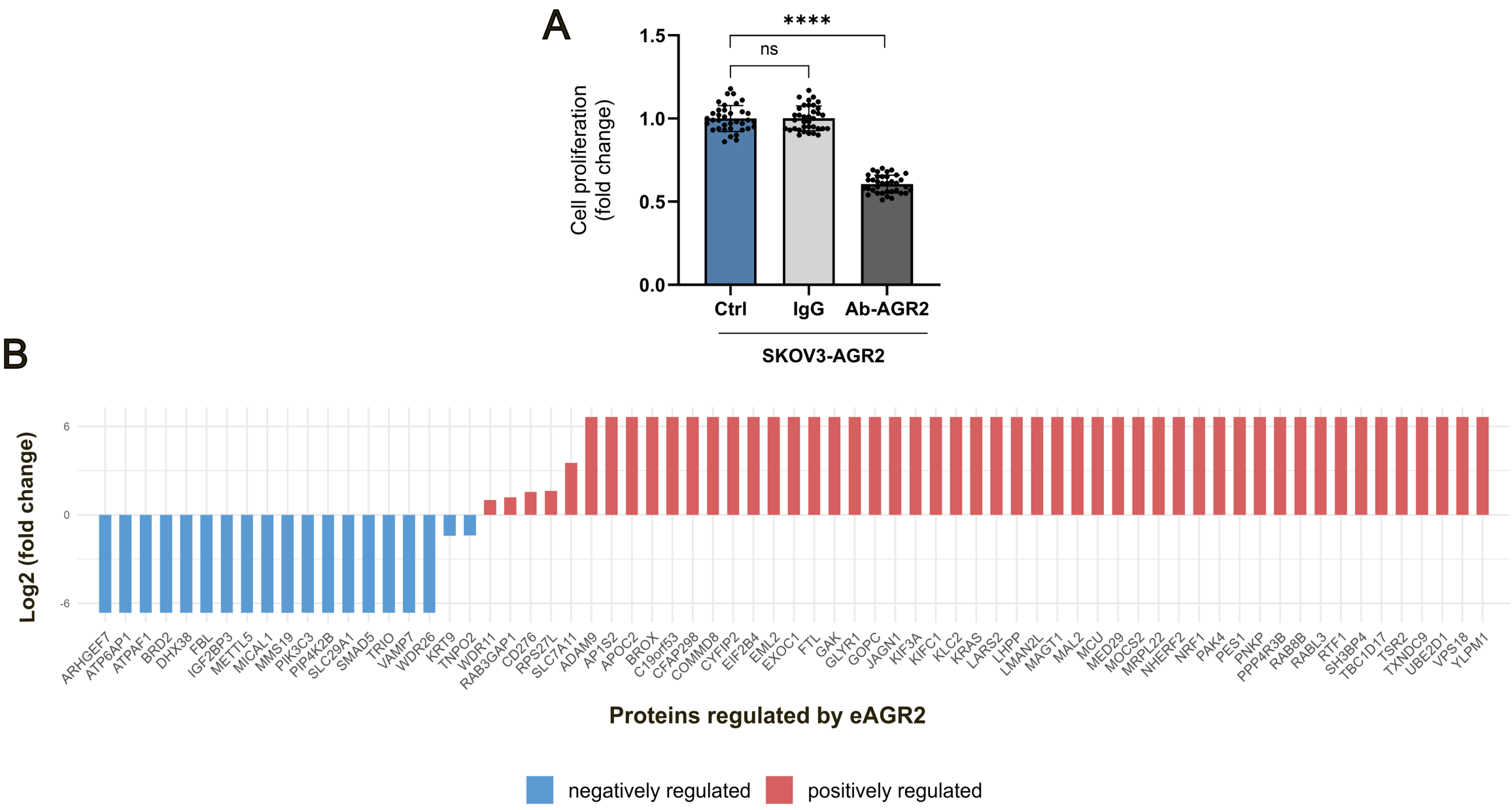

C

Source	Name	GO id	Adjusted p value	-log10(p.adjust)
GO:BP	nitrogen compound transport	GO:0071705	3.58E-04	3.45
GO:BP	vesicle-mediated transport	GO:0016192	2.07E-03	2.68
GO:BP	protein transport	GO:0015031	2.55E-03	2.59
GO:BP	organelle organization	GO:0006996	7.05E-03	2.15
GO:BP	macromolecule localization	GO:0033036	1.13E-02	1.95
GO:BP	establishment of protein localization	GO:0045184	3.24E-02	1.49
GO:BP	protein localization	GO:0008104	3.52E-02	1.45
GO:BP	cellular macromolecule localization	GO:0070727	3.77E-02	1.42
GO:BP	cellular component organization or biogenesis	GO:0071840	4.92E-02	1.31

D

Source	Name	GO id	Adjusted p value	-log10(p.adjust)
GO:CC	clathrin-coated vesicle	GO:0030136	1.39E-04	3.86
GO:CC	coated vesicle	GO:0030135	1.39E-04	3.86
GO:CC	autophagosome	GO:0005776	3.54E-03	2.45
GO:CC	lysosomal membrane	GO:0005765	5.61E-03	2.25
GO:CC	lytic vacuole membrane	GO:0098852	5.61E-03	2.25
GO:CC	vacuolar membrane	GO:0005774	8.66E-03	2.06
GO:CC	trans-Golgi network transport vesicle	GO:0030140	8.66E-03	2.06
GO:CC	kinesin complex	GO:0005871	1.52E-02	1.82
GO:CC	autolysosome	GO:0044754	2.68E-02	1.57
GO:CC	microtubule associated complex	GO:0005875	4.20E-02	1.38
GO:CC	secondary lysosome	GO:0005767	4.77E-02	1.32
GO:CC	phagocytic vesicle membrane	GO:0030670	4.77E-02	1.32

Supplemental Figure 5 - Proteomic analysis of proteins modulated by extracellular AGR2 (eAGR2).

(**A**) Validation of experimental conditions prior to proteomic analysis. SKOV3-AGR2 proliferation was monitored to confirm the efficacy of AGR2 inhibition by the blocking antibody before proceeding with mass spectrometry. Data are presented as mean ± SD from three independent experiments. Data are mean ± SD from three independent experiments; p-values were calculated using a two-tailed unpaired Student's t-test (*ns* = not significant, **** $p < 0.0001$). (**B**) Log2 fold change in abundance of 69 candidate proteins modulated by eAGR2 in SKOV3 cells. Proteins are ranked by fold change. Red bars indicate proteins downregulated in response to eAGR2, while blue bars indicate proteins upregulated. (**C, D**) Gene Ontology enrichment analyses were performed on significantly modulated proteins. Enrichment was assessed for Biological Process (BP) and Cellular Component (CC) categories based on the GO database, with adjusted $p < 0.05$ considered significant.

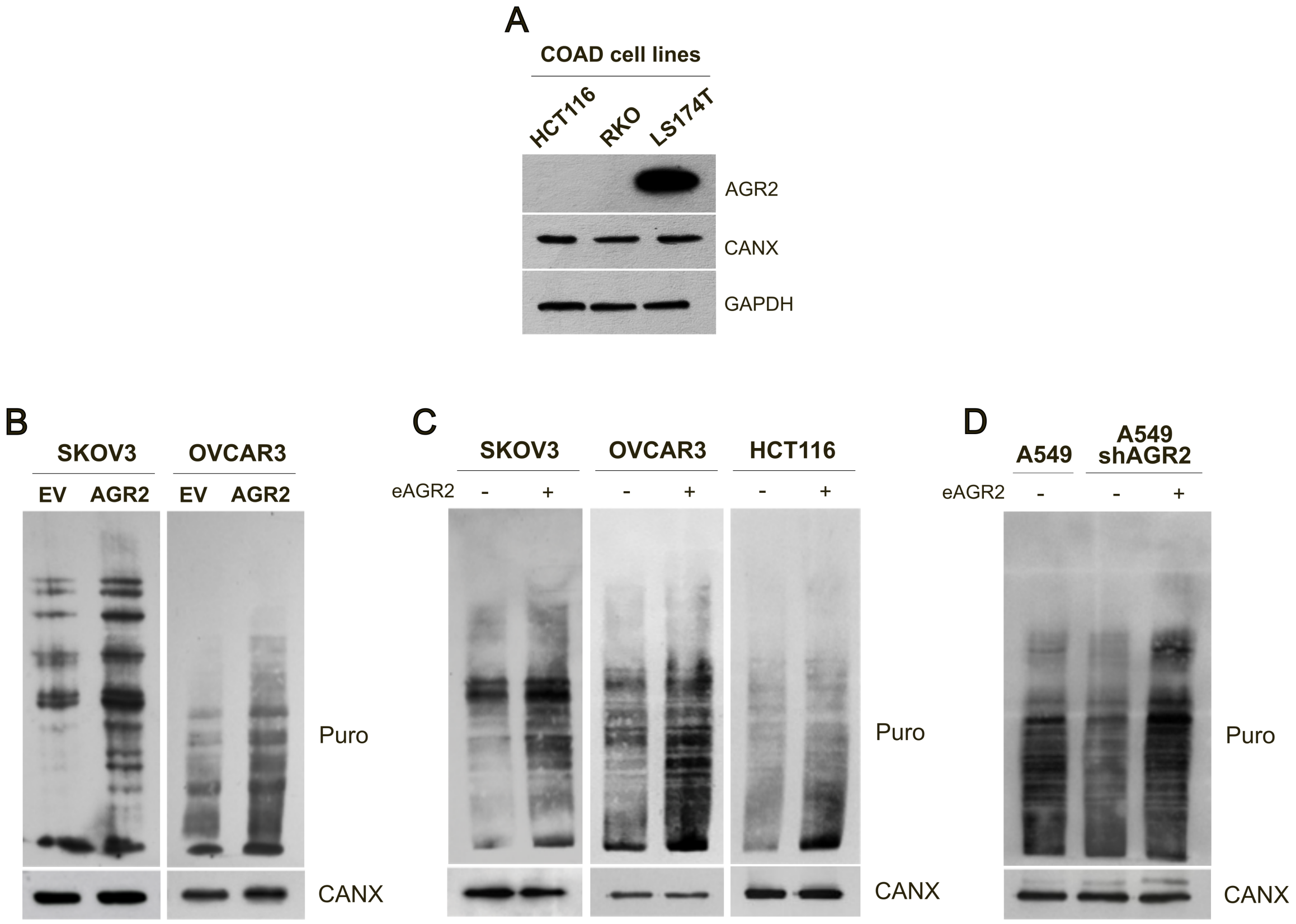

Supplemental Figure 6 - Extracellular AGR2 promotes global protein synthesis in different tumour cell lines.

(**A**) Western Blot analysis of AGR2 expression in colorectal cancer (COAD) cell lines, GAPDH served as the loading control. One representative experiment (n = 3) is shown. (**B**–**D**) Puromycin incorporation assays assessing global protein synthesis across different cellular models. (**B**) SKOV3 and OVCAR3 cells stably expressing AGR2 compared with empty vector (EV) controls. (**C**) OVCAR3, SKOV3, and HCT116 cells stimulated with recombinant AGR2 versus untreated controls. (**D**) A549 cells transfected with AGR2-specific shRNA (sh-AGR2) compared with control cells, cultured in the presence or absence of recombinant AGR2. In all experiments, increased puromycin signal reflects enhanced global protein synthesis in response to AGR2 expression or recombinant AGR2 stimulation. Calnexin and Ponceau Red staining served as loading controls. One representative experiment (n = 3) is shown.

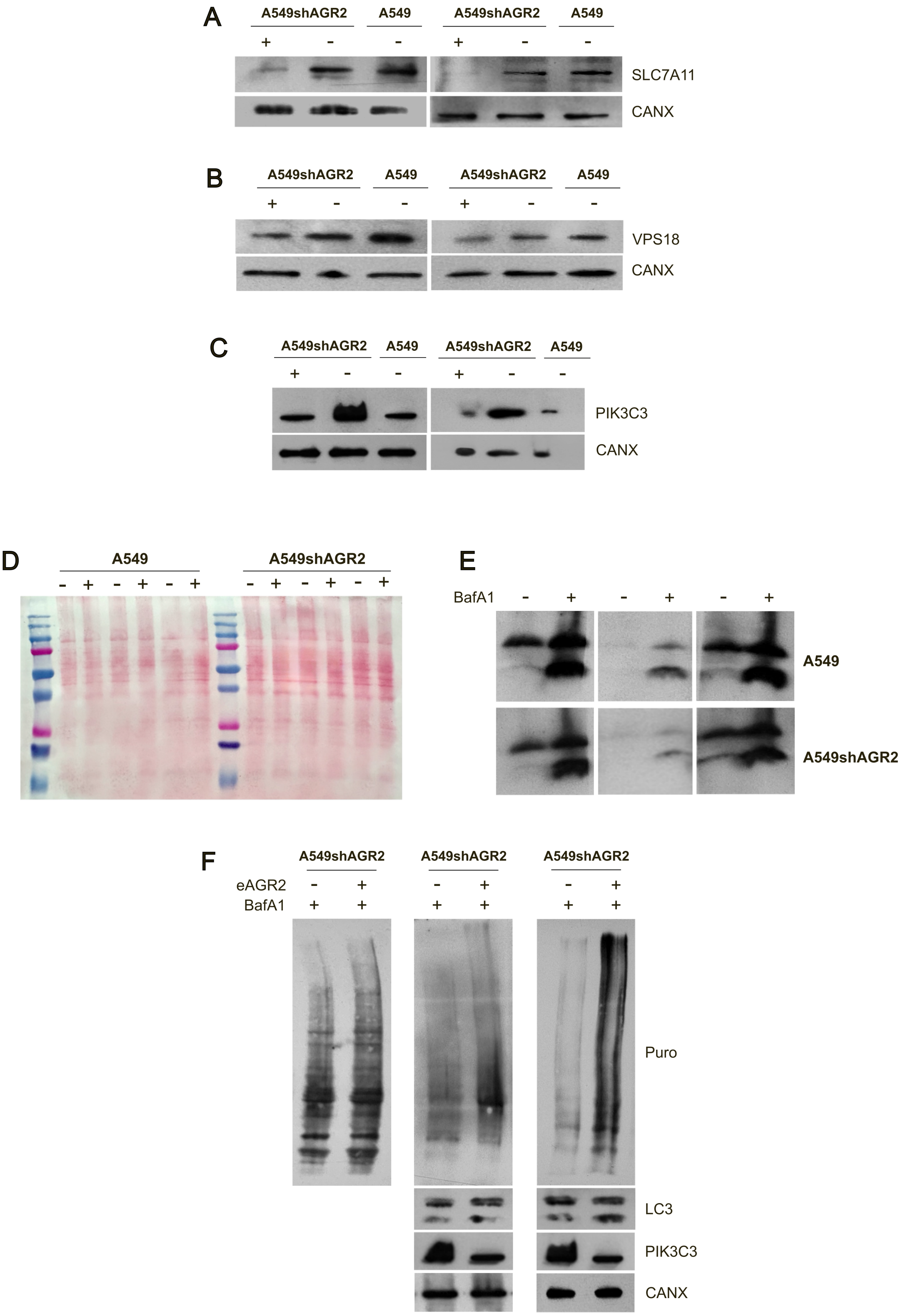
A
A549shAGR2
A549
A549shAGR2
A549
+ - - + - -
SLC7A11
CANX
B
A549shAGR2
A549
A549shAGR2
A549
+ - - + - -
VPS18
CANX
C
A549shAGR2
A549
A549shAGR2
A549
+ - - + - -
PIK3C3
CANX
D
A549
A549shAGR2
- + - + - + - + - + - +
E
BafA1 - + - + - +
A549
A549shAGR2
F
A549shAGR2
A549shAGR2
A549shAGR2
eAGR2 - + - + - +
BafA1 + + + + + +
Puro
LC3
PIK3C3
CANX

Supplemental Figure 7 – Additional replicates and loading controls for autophagy and protein synthesis assays.

(**A–C**) Additional replicate Western Blot images for SLC7A11 (**A**), VPS18 (**B**), and PIK3C3 (**C**) in A549shAGR2 cells treated with recombinant AGR2 (200 ng/mL) compared with untreated A549shAGR2 and parental A549 controls. CANX served as a loading control. (**D**) Ponceau S staining of membranes used for LC3 Western Blot analysis in A549 and A549shAGR2 cells treated with or without Bafilomycin A1 (BafA1; 150 nM), confirming equal protein loading across conditions. (**E**) Western Blot analysis of LC3 in A549 and A549shAGR2 cells treated with or without BafA1 (150 nM). Three independent experiments are shown. (**F**) Representative Western Blot images of puromycin incorporation (SUnSET assay), LC3, PIK3C3, and CANX in A549shAGR2 cells treated with BafA1 (150 nM) in the presence (+) or absence (–) of recombinant AGR2 (eAGR2, 200 ng/mL). Three independent experiments are shown.
